# Automated detection, labelling and radiological grading of clinical spinal MRIs

**DOI:** 10.1038/s41598-024-64580-w

**Published:** 2024-07-01

**Authors:** Rhydian Windsor, Amir Jamaludin, Timor Kadir, Andrew Zisserman

**Affiliations:** Visual Geometry Group, Department of Engineering Science, University of Oxford, Oxford, UK

**Keywords:** Software, Diagnosis, Medical imaging

## Abstract

Spinal magnetic resonance (MR) scans are a vital tool for diagnosing the cause of back pain for many diseases and conditions. However, interpreting clinically useful information from these scans can be challenging, time-consuming and hard to reproduce across different radiologists. In this paper, we alleviate these problems by introducing a multi-stage automated pipeline for analysing spinal MR scans. This pipeline first detects and labels vertebral bodies across several commonly used sequences (e.g. T1w, T2w and STIR) and fields of view (e.g. lumbar, cervical, whole spine). Using these detections it then performs automated diagnosis for several spinal disorders, including intervertebral disc degenerative changes in T1w and T2w lumbar scans, and spinal metastases, cord compression and vertebral fractures. To achieve this, we propose a new method of vertebrae detection and labelling, using vector fields to group together detected vertebral landmarks and a language-modelling inspired beam search to determine the corresponding levels of the detections. We also employ a new transformer-based architecture to perform radiological grading which incorporates context from multiple vertebrae and sequences, as a real radiologist would. The performance of each stage of the pipeline is tested in isolation on several clinical datasets, each consisting of 66 to 421 scans. The outputs are compared to manual annotations of expert radiologists, demonstrating accurate vertebrae detection across a range of scan parameters. Similarly, the model’s grading predictions for various types of disc degeneration and detection of spinal metastases closely match those of an expert radiologist. To aid future research, our code and trained models are made publicly available.

## Introduction

Back pain is the most common cause of long-term disability; for example, it affects around 80% of people in the UK during their lifetime^1^. As people live longer, incidence will only increase. Currently, due to the increased demand for radiological reporting across all parts of medicine, radiological imaging is a major bottleneck in back pain diagnosis and care management. We aim to combat this by developing a complete computer aided detection and diagnosis tool to assist radiologists by helping diagnose and monitor aetiology such as degenerative changes. Our objective is to offer a completely automated set of tools for performing common gradings & measurements in clinical spinal MR scans in a manner that is quick, effective and cheap to perform. Such technology has multiple use cases: (1) triaging scans such that severe cases can be reviewed immediately by clinicians; (2) acting as a decision support tool for radiologists; (3) enabling larger scale studies into the underlying causes of back pain where the number of scans that can be manually annotated is a limiting factor. There are several challenges associated with this task. For example, clinical spinal MR scans show a great deal of variation depending on several factors, including: scan protocol, field of view, MR machine used, noise from external sources such as movement or surgical implants and large variations in subject anatomy, either naturally (e.g. scoliosis, transitional vertebrae, trauma fractures) or through prior clinical treatment (e.g. radiography, surgery). Any system ready to be clinically deployed must be robust to these ‘in-the-wild’ variations which we consider in this work.

**Figure 1:**
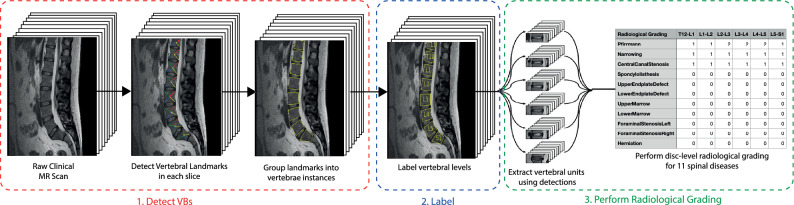
An overview of our end-to-end pipeline, shown grading intervertebral discs for degenerative changes on a T2w lumbar MR scan. Note that the detection and labelling pipeline works across a range of different MR sequences (T1w, T2w, STIR, etc.) and fields-of-view (e.g. cervical, thoracic, lumbar and whole spine). In this paper we also investigate how our approach can be used to detect spinal metastases, vertebral fractures and cord compression.

This paper introduces an end-to-end system that takes in spinal MR scans as input, and outputs radiological gradings not unlike those seen in radiological reports. The description of the system is broken into two sections: the first describes the method to detect and label vertebral bodies in an MR scan; and the second describes the method for performing radiological grading of vertebral body and intervertebral disc degenerative changes, and other spinal conditions including spinal metastases, cord compression and vertebral fractures. This two-stage process is illustrated in Fig. 1, shown performing radiological grading of a lumbar MR scan for disc degenerative changes.

Our work expands on two separate Medical Image Computing and Computer Assisted Intervention (MICCAI) conference papers; Windsor et al. 2020^2^, which introduced a novel CNN-based vertebrae detection and labelling pipeline, and Windsor et al. 2022^3^, which introduced a transformer-based automated radiological grading framework. The extensions include: (i) marrying together these two methods into one single end-to-end system that operates without human intervention, taking sagittal spinal MR scans as input and outputting radiological gradings on a per-intervertebral level basis; (ii) additional explanation of the methods, particularly on handling scans with varying fields of view, and adding robustness to our detection pipeline at test time, specifically handling missed detections; (iii) additional results by comparing to another expert grader on the spine cancer tasks; and (iv) publicly releasing the code and trained model weights for academic use by clinical and computer vision researchers: https://github.com/rwindsor1/SpineNet. This pipeline has also been validated on multiple external clinical datasets and has been found to have comparable performance to expert radiologists and physicians^4,5^; the detection and labelling portion of the pipeline has also been used as-is on whole spine MRIs while the classification pipeline has been fine-tuned for Ankylosing Spondylitis detection^6^.

### Related work

This work can be viewed as the follow-up to SpineNet, with several improvements over the initial version^7^. Firstly, the initial version relied on a deformable part model with histograms-of-oriented-gradients (HOG) templates to detect vertebral bodies (VBs) and a parts-based graphical model for assigning labels to the detected VBs (e.g. S1, L5, L4, etc.)^8,9^. This has been replaced by a fully deep-learning based approach that is significantly more accurate and is able to deal with a range of fields of view (i.e. lumbar, thoracic, cervical and full spine scans), and different MR sequences (e.g. T1w, T2w, STIR). Furthermore, the new detection stage is considerably faster taking only a few seconds to detect and label all VBs in a single lumbar scan as opposed to around a minute as in the original version. Secondly, the network used for radiological grading has been improved. The original SpineNet used a VGG-F network operating on single volumes encompassing intervertebral discs to perform a range of classification tasks. In our work, this grading network is replaced by a new architecture; the Context-Aware Spinal Transformer (CAST). This model operates on an entire sequence of intervertebral discs simultaneously, allowing contextual information from neighbouring vertebrae to be incorporated into grading predictions. Empirically, we find that this improves agreement with expert radiological gradings and allows for multiple MR sequences to be used when grading.

Detecting and labelling of vertebrae in medical images has been a long-standing task in medical imaging due to its importance to a range of diagnosis tasks. Initial approaches used a variety of methods such as HOG-based templates^8,9^, supervised classification forests^10,11^ and deformable models^12,13^. However, in recent years deep learning has become the standard approach, with applications across multiple modalities such as CT, MR and X-ray^14–21^.

Other works have also used deep learning to classify spinal disorders, however, these are generally limited to single-task models (e.g. stenosis^22^, disc herniation^23^ and Pfirrmann grading^24,25^). In terms of spinal cancer, to our knowledge two works train models to detect metastases based on 2D images extracted from MR scans^26,27^ and Merali et al.^28^ propose a model to automatically detect metastatic cord compression. Another related work is Lemay et al.^29^, who train a model to segment spinal cord tumours. However, these approaches are all restricted to single tasks and not vertebral body metastases, fractures and cord compression detection as attempted here.

## Detecting and labelling vertebral bodies

Since the spinal disorders considered in this paper pertain to individual vertebrae or intervertebral discs, the first stage of the proposed approach is to detect each vertebral body such that individual vertebrae or discs can be localised. This is done in two steps; firstly each vertebral body is detected in 3D. Given this sequence of detections, a *labelling* stage then assigns levels to each vertebra detection (e.g. S1, L5, T3, C7, etc.). Our proposed method is an extension of that originally introduced in our initial conference paper^2^. and accordingly some description of the method is adapted from that work.

**Figure 2:**
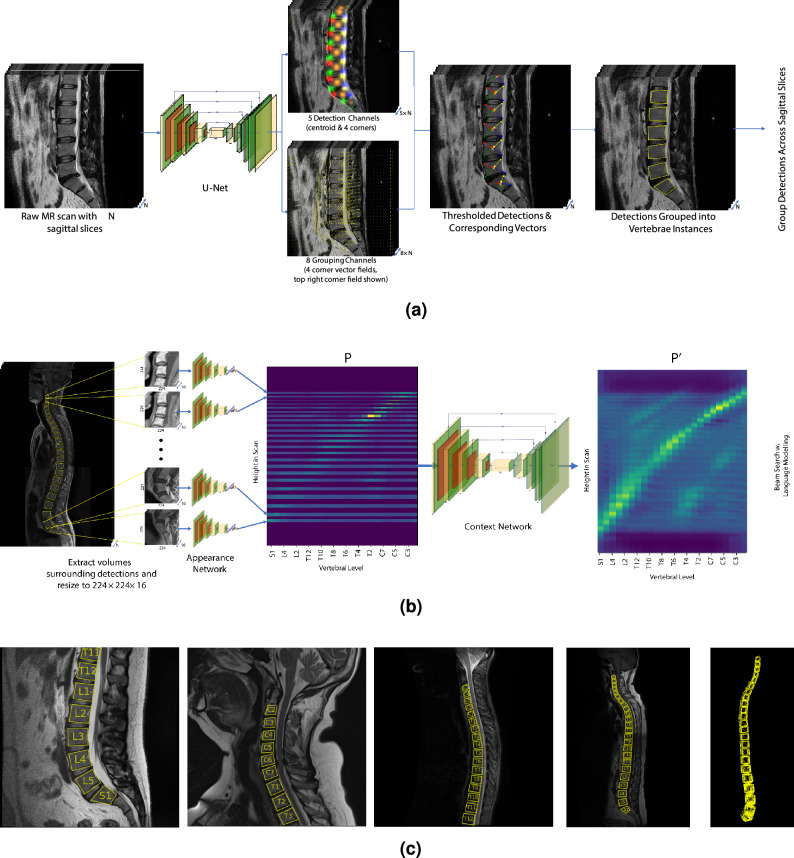
The detection and labelling process used in our experiments with example results for a range of clinical scans. (**a**) The Vector Field Regression method used to detect vertebral bodies in scans. The U-Net has 13 output channels—5 which regress gaussian peaks over the vertebral landmarks (corners and centre) and 8 corresponding to the *x* and *y* components of 4 vector fields for each corner. These are then grouped to produce a series of quadrilaterals in each slice. (**b**) The language modelling-inspired approach to vertebra labelling. A probability-height map, *P*, is constructed based on appearance information alone. This map is refined by a context network, resulting in *P*′. *P*′ is then decoded into a valid sequence of levels via a penalised beam search. (**c**) Example vertebral detections with corresponding predicted level labels. The far-right panel shows a 3-D reconstruction of the neighbouring spine based on detections.

### Vertebral body detection by vector field regression

The first stage of our approach is to detect vertebral bodies (VBs) in the raw scans. To achieve this, we introduce a method called *Vector Field Regression* (VFR), illustrated in Fig. 2a. In this approach, vertebrae are represented as a series of quadrilaterals across sagittal slices in which the vertebra is visible. Firstly, a U-Net^30^ style model operates on each sagittal slice (or patch of a slice). This network outputs Gaussian heatmaps centred on the centroids and corners of each visible VB. In total, this U-Net has 5 output detection channels; one for each corner type and one for the VB centroid. One can then apply local maximal suppression to these output heatmaps to detect points of each landmark type. However, this is not sufficient to detect vertebral bodies; in addition to detecting these landmarks, one must also be able to group them into individual vertebral instances. Accordingly, for each type of corner detected, a corresponding vector field is also output. At the point of each corner, this corresponding vector field should ‘point’ to the centroid of the vertebra to which it belongs. This allows landmarks from the same vertebra to be grouped together in a simple manner which is robust to rotations, flips and variable vertebra size. Accordingly, 8 additional ‘grouping’ channels are added to the U-Net (for the *x* and *y* coordinates of the vector field for each of the corners). These landmarks and the corresponding vector fields are then sufficient to produce vertebral detections by simply looping through each detected centroid and then finding the four corner landmarks (one of each type) which ‘point’ closest to it.

At this point, each vertebral detection consists of a centroid and four corners, together forming a quadrilateral in an individual sagittal slice. The next step is to group 2D quadrilaterals corresponding to the same vertebra across sagittal slices, forming a single 3D volume for each vertebra. This is accomplished by measuring the intersection-over-union (IOU) of quadrilaterals in neighbouring slices. If this is large, the quadrilaterals are grouped together into a single vertebral instance. Starting from the slice with the most vertebral detection and moving out to the left and right, 3D volumes across slices are constructed corresponding to each visible vertebra. Example results are shown in Fig. 2c.

**Splitting Large Scans Into Patches.** Larger non-square scans (such as whole spine scans) are split into patches before VFR is applied. This is done by splitting the scan into a grid of overlapping squares with an edge length of 50cm (determined by the pixel spacing parameter in the DICOM header) and an overlap of 40% between neighbouring patches. This specific value was chosen experimentally and found to be a good trade-off between speed (larger overlaps increased the number of inferences the detection model must make) and accuracy (smaller overlaps increase the likelihood of missed landmarks because of them being only partially visible at the edge of patches). However we also found the method was fairly robust to variations in this overlap value.

The output from the detection and grouping channels are used to find landmarks in each patch. These landmarks are then transformed back into the frame of the original scan. At this point, the algorithm proceeds as before by grouping landmarks into slicewise polygons and then across sagittal slices. The process of patch-splitting for a whole spine scan is shown in the supplementary material. Patch size is chosen to be approximately the same as the field of view for a typical lumbar MR scan ($25\text {cm}^2\times 25\text {cm}^2$).

**Robust Vector Field Regression.** It is possible that some landmarks for certain vertebrae are missed in the initial detection stage. For example, some vertebrae may be collapsed and no longer have a clear quadrilateral appearance. In such cases, where a centroid is detected with three corner detections which point closely to it, the approximate location of the fourth corner can be estimated by simply adding the pointing vector of the opposite corner to the centroid point. Similarly, if four corner detections are left unassigned to vertebrae but point to a similar location, a centroid can be inferred at that point. In practice, we find this slightly improves the performance of our detection system on subjects with extreme pathology although is rarely necessary for ordinary clinical scans.

### Convolutional labelling of vertebral levels

The VFR method described above allows us to detect vertebral bodies in a sagittally sliced MR scan. The next step of the pipeline is to determine which vertebral levels these detections correspond to (e.g. L4, L5, S1 etc). This is made more challenging by the fact that we are not constrained to a single field of view. As such, ‘counting up’ methods, i.e. those that rely on an anchor vertebra being visible (such as C2/S1 at the top/bottom of the vertebral column) are unsuitable. Furthermore, such methods are not robust to missed detections or variations in the number of vertebrae in the vertebral column (e.g. in cases where a transitional vertebra is present).

There are two pieces of information to consider when labelling a vertebra - its *appearance* (e.g. intensity pattern, shape, size etc.) and its *context* (the vertebra’s position relative to other detections in the scan). For example, S1 usually has a very distinctive shape which allows it to be labelled from appearance alone. On the other hand, L5 looks very similar to other lumbar vertebrae, however, can be easily identified from its context - it is the next vertebra up from S1. Our method attempts to use both of these sources of information when labelling a vertebra.

Firstly, a 3D volume around each detected VB is fed as input to an *appearance network*. This outputs a 23-element (from C3 to S1) probability vector as an initial prediction of the level of the VB based on its appearance alone. We do not include C1, C2, and the remainder of sacrum and coccyx since these are either hard to see or their shape is not easily approximated as a quadrilateral in sagittal MR slices and thus often not detected. Once the appearance network’s initial predictions for each VB level have been made, the next step is to incorporate information about the spatial configuration of the detections. Accordingly, a probability-height map, *P* is constructed of dimension $H\times 23$, where *H* is the height of the scan in the superior-inferior axis and 23 represents the outputs of the appearance network. At the height of each detection, *P* has a value equal to the output from the appearance network for that detection. Using this as input, a convolutional *context network* refines this probability-height map, taking into account appearance predictions from spatially proximal VBs to update the probability vectors for each detection. The result is a refined map $P'$, as is shown in Fig. 2b.

The final step is to decode $P'$ into discrete level predictions. Naïvely, this could be done by taking the maximum probability level at the height of each detection in $P'$. However, this would allow for nonsensical outputs, such as the same level for two detections. Ideally, we also want to build in soft constraints such that successive detections are labelled as successive labels. For example, we would expect S1 to be the detection below L5. However, we also wish to remain robust to missed detections.

To build these constraints into our approach, we take inspiration from language modelling. Using a beam search, we can find the most probable valid sequence of levels for the detections. Penalties are added to the sequences probability score in the case of transitional vertebrae or numerical variations to reflect the unlikeliness, yet a possibility, of such events.

At this point, each vertebra from S1 to C3 which is visible in the scan should have been detected and assigned a level label. These detections can be used in a range of applications. For example, the detections can be used to measure spinal curvature (for scoliosis diagnosis and monitoring) or extract regions of anatomical interest to be fed to a downstream classification network (e.g. intervertebral discs or regions surrounding vertebral bodies, perhaps for classifying degenerative changes or spinal metastases respectively).

### Datasets

The capability of our detection system is assessed on three separate datasets; **Oxford Whole Spine (OWS)**, **Genodisc** and **Zukić**. In all cases data identification, anonymisation, pre-processing and experiments are carried out in accordance with relevant guidelines and regulations from the UK Health Research Authority. Each dataset consists of a multiple spinal MR scans distributed in DICOM format.

**OWS** is a dataset of 710 anonymised full spine MR scans across 196 patients extracted from the Oxford University Hospitals NHS trust. This dataset is a part of Oxford Secondary Care Lumbar Magnetic Resonance Imaging Cohorts (OSCLMRIC), a UK Health Research Authority approved study (IRAS Project ID 207858). Since scans are anonymised, the need for informed consent from patient was waived. These scans are distributed across a range of commonly used clinical sequences (mostly T1w, T2w and STIR). Each vertebral body from S1 to C2 is annotated as a quadrilateral in the central slice of each scan by a non-specialist. This dataset is split into training, validation and testing datasets 80%:10%:10% respectively down the patient line.

**Genodisc** is a large dataset of lumbar MR scans, containing 2287 studies with sagittal scans, with subjects recruited from multiple clinical imaging centres across Europe. This dataset was curated by the Genodisc Consortium with UK Research Ethics Committee approval 09/H0501/95. Equivalent IRB/REC approvals were obtained in each recruiting country. Each vertebra from S1 to T12 is annotated as a series of polygons across sagittal slices. Each scan was also labelled for multiple spinal degenerative changes by an expert radiologist, which we use in "Automated grading of disc degenerative changes, spinal metastases and related conditions" section. We split this dataset down the patient line, with 1880, 203 and 204 studies in the training, validation and test sets respectively. Further details the approval of for **OWS** and  **Genodisc** are given in Section 7.

Finally, **Zukić**^31^ is a publicly available dataset of mostly lumbar sagittal MR scans from 17 subjects available on the online SpineWeb platform (http://spineweb.digitalimaginggroup.ca/). This dataset is used for testing only.

### Training and implementation

There are 3 constituent neural networks in the detection and labelling pipeline; (i) the VFR regression network, (ii) the appearance network, and (iii) the context network. The VFR model is a ResNet18-encoded^32^ UNet^30^. The appearance network is a simple VGG-F convolutional neural network^33^. Finally, the context network is a conventional UNet^30^. All networks are implemented in PyTorch.

**Training the Vector Field Regression network.** The model to regress vector field (as shown in Fig. 2a) is trained on patches of sagittal slices from the training splits of both the **Genodisc** and **OWS** datasets. Patches are resampled to 224 pixels via bicubic interpolation. Ground truths are constructed from VB quadrilaterals marked by annotators. Targets for the detection and grouping channels are generated as follows: For the detection channels, gaussian peaks are added to the corresponding channels centered on each vertex and on the centroid. These peaks have a maximum value of 1 and variance proportional to the quadrilateral’s surface area. The grouping channels for each vertex are constructed such that, for an area around each vertex proportional to the VB’s surface area, the two corresponding grouping channels represent a vector field which points to the VB’s centroid.

Once this target tensor, ${\hat{Y}}$, is constructed, the detection network is trained end-to-end using the following composite loss function for output tensor *Y*; ${\mathscr {L}}(Y,{\hat{Y}}) = {\mathscr {L}}_{detect}(Y,{\hat{Y}}) + {\mathscr {L}}_{group}(Y,{\hat{Y}})$. An L1-regression loss is applied to the detection channels,

1 $$\begin{aligned} {\mathscr {L}}_{detect}(Y,{\hat{Y}}) = \sum _{k=1}^{5}\alpha _{ijk}\left| y_{ijk}-{\hat{y}}_{ijk}\right| , \end{aligned}$$

where *k* indexes the landmark channel (the four VB corners and centroid), (*i*, *j*) indexes the position in the patch and $\alpha _{ijk}$ is a weighing factor given by

2 $$\begin{aligned} \alpha _{ijk}= {\left\{ \begin{array}{ll} \frac{N_k}{N_k+P_k} &{} \text{ if } {\hat{y}}_{ijk} \ge T\\ \frac{P_k}{N_k+P_k} &{} \text{ if } {\hat{y}}_{ijk} < T \end{array}\right. } \end{aligned}$$

with $N_k$ and $P_k$ being the number of pixels in the target detection channel respectively less than or greater than some threshold *T* ($T=0.01$ in this case). The vector field grouping channels are supervised by an L2-regression loss;

3 $$\begin{aligned} {\mathscr {L}}_{group}=\sum _{l=1}^4\sum _{b}\sum _{(i,j)\in {\mathscr {N}}_{bl}}||{\textbf{v}}_{ij}^{l}-{\textbf{r}}_{ij}^{k}||_2^2. \end{aligned}$$

Here *l* indexes each corner type/vector field, *b* indexes the annotated VBs in the patch and ${\mathscr {N}}_{bl}$ is a neighbourhood surrounding the $l^{th}$ corner of the $b^{th}$ VB annotated in that patch. ${\textbf{v}}_{ij}^l$ is the value of the output vector field corresponding to corner *l* at location (*i*, *j*) and ${\textbf{r}}_{ij}^b$ is the ground truth value of the vector field, i.e. the displacement vector from the centroid of VB *b* to location (*i*, *j*). We use a square neighbourhood, ${\mathscr {N}}_{bl}$, with edge length of $\frac{A_b}{3}$ where $A_b$ is the size of the target quadrilateral for VB *b*. We use heavy augmentation during training including image rotation, rescaling and flipping in the coronal plane. The network is trained using an Adam optimizer with a learning rate $10^{-3}$ and $\mathbf {\beta }=(0.9,0.999)$.

**Labelling with Appearance and Context.** Both the appearance and context networks are trained on the **OWS** dataset only since it contains whole spine scans and thus a balance of vertebral level labels (as opposed to the **Genodisc** and **Zukić** datasets, which are predominantly lumbar scans). This is done as follows: Firstly a volume is extracted around each annotated VB. This is done by tightly fitting a bounding cuboid around each detection and then expanding the box by 50% in each direction to capture nearby anatomical structures. The resulting volume is then resampled to a size of $224 {\hspace{-1.111pt}\times \hspace{-1.111pt}}224 {\hspace{-1.111pt}\times \hspace{-1.111pt}}16$ voxels (isotropically along the axial and coronal axes but not sagittally). These volumes are then given as input to the appearance network which attempts to classify the vertebra from C3 to S1 in a 23-way classification problem. This network is trained using a standard cross-entropy loss.

The context network is also trained as follows. Input height-probability maps are constructed such that for a given detected VB, with height calculated from $y_a$ to $y_b$, with centroid at $y_c=\frac{y_a+y_b}{2}$, the height-probability map *P* has the same value as the temperature-softmaxed (T=0.1) predictions from the appearance network from height $y_c - 0.5\times (y_b-y_a)$ to $y_c + 0.5\times (y_b-y_a)$. The context network is an image-to-image translation network which takes *P* as input and then outputs a refined version of *P*, denoted $P'$. $P'$ is then decoded into a discrete series of predictions at the height of each VB detection using a beam search. A visual representation of this process is shown in Fig. 2b. As an augmentation during training each vertebra detection is dropped from *P* with a probability of 0.1. A loss function is still applied to the predictions at height of the missing detection, on the basis that the model should be able to infer the vertebra’s level from the surrounding detections. Both the appearance and context networks are trained using an Adam optimizer with a learning rate of $10^{-3}$ with $\mathbf {\beta }=(0.9,0.999)$.

### Results

The results of detection and labelling on all datasets are shown in Table 1 with comparisons to other methods reported on the same datasets where available. Our convolutional labelling pipeline is also compared to a simple LSTM^34^ labelling method which takes features from the appearance network alone as input (sorted by the height of the detection in the scan) and predicts the levels of the detection sequence. To account for **Zukić**, where a single point marks the centre of each vertebra rather than a complete quadrilateral, a correct detection is deemed to be correct when the annotated centroid is contained entirely within exactly one detected quadrilateral of the same slice. To measure the performance of the detection stage of the pipeline, we report precision and recall under such a definition, as well as the *localisation error* (LE), the distance of the detected quadrilateral centroid from the ground truth annotation in millimetres. To assess the quality of vertebral labelling we report the identification rate (IDR) - the fraction of vertebrae detected and labelled correctly. To account for cases where the entire sequence is shifted by one vertebra, perhaps due to a transitional vertebra, we also report the the fraction of correctly detected vertebrae which are labelled within one level of the ground truth (IDR$\pm 1$).

**Table 1:** The results of our method for detecting and labelling vertebrae when applied to multiple datasets of clinical MR scans.

Dataset	# Scans	# Vertebrae	Method	Precision (%)	Recall (%)	IDR(%)	IDR± 1(%)	LE (mm)
**OWS** (Whole Spine)			Windsor^†^^17^	99.4	99.4	–	–	1.0 ± 0.9
	37	888	Label baseline	–	–	86.9	93.4	–
			Ours	99.0	98.1	96.5	97.3	2.4 ± 1.3
**Genodisc** (Lumbar)			Lootus^8^	–	–	86.9	–	3.5 ± 3.3
	421	2947	Label Baseline	–	–	90.1	97.4	–
			Ours	99.7	99.7	98.4	99.7	1.6 ± 1.1
**Zukić** (Lumbar)			Zukić^31^	98.7	92.9	–	–	1.6 ± 0.8
	17	154	Label Baseline	–	–	87.0	94.3	–
			Ours	99.3	98.7	90.9	98.7	2.0 ± 1.5

IDR and LE indicate the identification rate and localisation error respectively. IDR$\pm 1$ indicates the identification to within one level of the ground truth. ^†^Please note that the previous method proposed in Windsor *et al.* 2020^17^ requires the bottom vertebrae in the scan to be manually annotated and prior knowledge of the number of vertebrae to detect. Therefore, it cannot be considered an entirely automated method.

As seen from Table 1, our detection system significantly improves on that of the original SpineNet^8^ for lumbar scans and generalizes well to whole spine scans. Qualitatively, we note that the system also performs well across a variety of fields-of-view and in various cases of spinal pathology. Examples of these effects are included in the supplementary material.

## Automated grading of disc degenerative changes, spinal metastases and related conditions

This section discusses the second stage of our proposed approach. That is, given vertebral detections and scan as input, the method to perform classification for a range of different spinal disorders. To perform this grading/classification, we utilize the Context-Aware Spinal Transformer architecture (CAST), which we initially introduced in our conference paper^3^. Therefore, some aspects of the method’s description and experiments presented here are adapted from our previous work. Note that the initial acroynm for model (SCT) has been changed to CAST to avoid confusion with the Spinal Cord Toolbox^35^, an existing open-source tool for analysing spinal cord MRI data.

**Figure 3:**
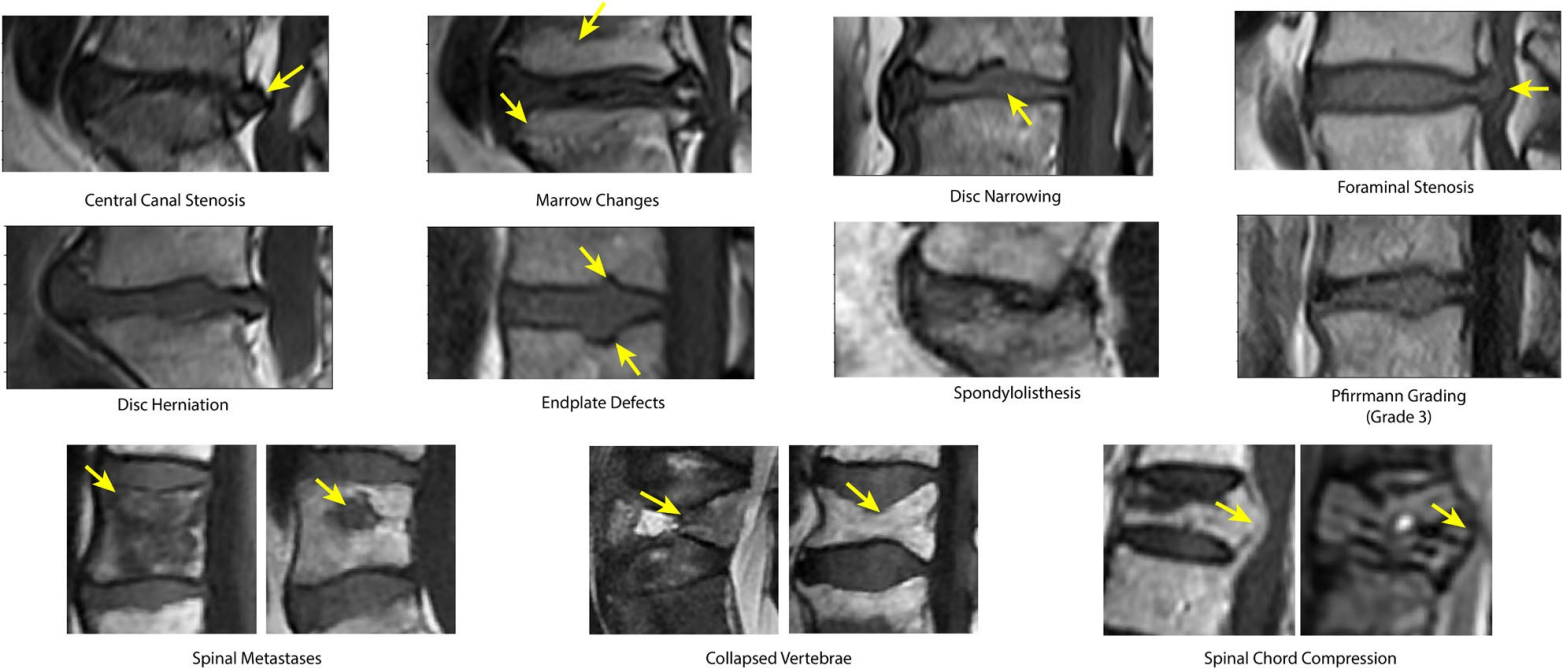
Spinal MR imaging analysis tasks considered in this paper. The top two rows show degenerative changes, graded for each intervertebral disc in the scan. The bottom row shows tasks related to spinal cancer, where predictions are made for each detected VB.

This architecture is applied to two tasks on separate datasets; (a) grading degenerative changes associated with intervertebral discs in T1w and T2w lumbar scans, and (b) detecting spinal metastases and the related conditions of cord compression and vertebral fractures (Fig. 3).

### Context-aware spinal transformer

Like other models used to assess vertebral disorders such as SpineNet^7,36^ and DeepSpine^22^, CAST extracts features from each vertebra in the spine using the same model. However, there are two important differences to these models.

**Figure 4:**
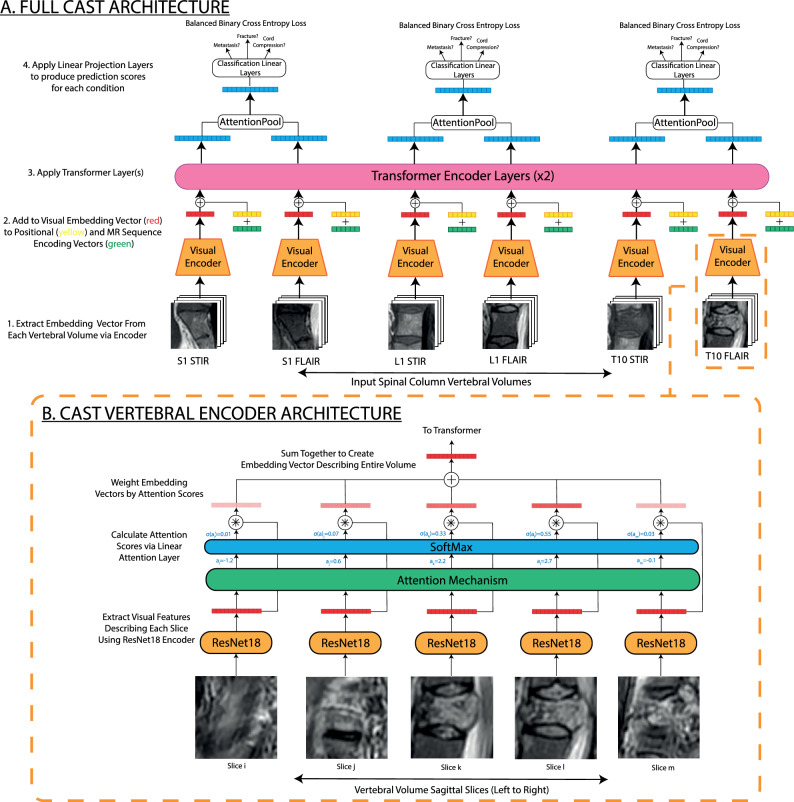
The Context-Aware Spinal Transformer (CAST) ingests a sequence of vertebra or intervertebral disc volumes and performs vertebra- or disc- level classification tasks. In this case CAST is shown operating on vertebral volumes to perform the cancer detection task, however it can be applied analogously to vertebral disc grading tasks. Subfigure A shows the full CAST architecture; this can be broadly separated into two stages—firstly an embedding vector describing each volume is output by the same vertebral encoder model, shown in subfigure B. These are then input into a transformer, allowing for contextual information to flow between the volumes and update the embedding vectors. STIR and FLAIR sequences are shown here, however, the disc grading task uses strictly T1w and T2w sequences, whereas the spinal cancer task uses a range of common sequences (T1w, T2w, STIR, FLAIR etc). Note that a key advantage of this architecture is that it can be applied to variable number of volumes without adaption.

First, instead of aggregating information across sagittal slices using a either max-pooling or 3D convolutions, a 2D ResNet18^32^ adapted for single-channel images extracts features from each slice. These are then reduced to a global embedding for the entire vertebra by a standard multi-headed attention mechanism (Fig. 4). There are multiple reasons why we believe this is a better architectural choice: (i) Applying the same convolution across sagittal slices implies a constant slice thickness between scans. However, this assumption is not justified in most clinical datasets, where different scan protocols will use different slice thicknesses. This can be somewhat mitigated by resampling scans in the sagittal axis during pre-processing, but this may introduce aliasing effects to the image. (ii) Without any adaption, the model can operate on both 2D and 3D scans. Furthermore, the ResNet18 model can be initialised with weights from large-scale pre-training on natural image datasets (e.g. ImageNet^37^) (iii) Sagittal slices are attention-weighted during inference, allowing the model to effectively ignore partial-volume effects often observed in peripheral slices. This attention weighting also allows easier interpretation of grading decisions, since the user can determine which slices were most influential in the model’s grading decision. The supplementary material contains an example of this phenomenon.

In addition to using attention to collate features across sagittal slices, it is also used to aggregate features extracted across multiple MR sequences. Output visual embeddings for each vertebra in each modality are passed to a 2-layer transformer encoder, added to learnt embeddings for the MR sequence and vertebral level (shown in yellow and blue in Fig. 4). Feature vectors corresponding to the same vertebra are then joined into a single representation by a final attention-pooling layer. For each grading task, predictions are generated by applying a specific linear layer to this embedding.

A significant advantage of this architecture is that it allows for multiple different MR sequences to be included at inference, however does not necessitate a specific set. For example, T1w only, T1w+T2w or T2w only can all be used without modifying the architecture. This is particularly useful for ‘in-the-wild’ clinical data, where different scanning protocols are used depending on the type of imaging investigation and the centre where the study is performed.

### Disc-level radiological grading of spinal degenerative disorders

In this section, we evaluate the proposed architecture on the task of grading degenerative changes in clinical lumbar MR scans. SpineNet^7,19^ performed this task using T2w clinical scans and we compare to this version here and introduce a new model which considers T1w & T2w scans together.

**Dataset.** We train and evaluate radiological grading performance on the **Genodisc** dataset (introduced in Sect. "Datasets"). Each vertebral disc in each scan from L5/S1 to T12/L1 is labelled by an expert radiologist for the following degenerative changes: Pfirrmann Grading (5 classes), Disc Narrowing (4 classes), Central Canal Stenosis (4 classes), Upper & Lower Endplate Defects (Binary), Upper & Lower Marrow Changes (Binary), Left & Right Foraminal Stenosis (Binary), Spondylolisthesis (Binary), Disc Herniation (Binary). In total the dataset consists of 2414/2316 T1w/T2w scans in the training dataset and 227/258 T1w/T2w scans in the test set. Almost all studies have a T1w and T2w scan together.

**Training and Implementation.** Once the vertebrae visible in a scan are detected, volumes surrounding anatomical volumes important to a given disorder can be extracted for analysis. In our case, we perform the classificatithis dataset down the patient line, with 1880, 203 and 204 studieson of spinal degenerative changes on the *intervertebral disc* level. To extract the disc volumes, the mid-point between the centroids of two consecutive VB detections is calculated, defining the centre. This volume is then rotated such that the lower endplate of the upper vertebra is horizontal (this angle is calculated using the lower side of the detection quadrilateral). The width of the extracted endplate volume is then defined as double that of the larger VB detection. The height of the disc volume is then chosen such that the aspect ratio of the extracted patch is 2:1. These volumes are then resampled to a size of $112{\hspace{-1.111pt}\times \hspace{-1.111pt}}224{\hspace{-1.111pt}\times \hspace{-1.111pt}}S$. Examples of these volumes can be seen in Fig. 3. All models are trained until 10 successive validation epochs do not result in a decreased loss. An Adam optimizer is used with a learning rate of $10^{-4}$ and $\mathbf {\beta }=(0.9,0.999)$. A batch size of 20 is used for the lumbar-only degenerative changes task and 6 for the whole-spine cancer task (each sample consists of each VB in each MR sequence of the scan). In total training for both tasks consumes around 40GB of GPU memory and takes approximately 6 hours using 2 Tesla P40s. The classification linear layers are then finetuned for each task for 10 epochs with the encoder and transformer weights frozen. Standard augmentations for each vertebra are used including rotation ($\pm 15^\text {o}$), translation ($\pm 32$px), scaling ($\pm 10\%$) and intensity augmentation ($\pm 10\%$). For the multi-sequence models trained on T1w and T2w sequences, a single sequence or both sequences are dropped during training with probabilities of 0.4 and 0.1 respectively, leaving the model to make predictions for a volume it has not seen based on context alone. 50% dropout is used for the transformer layers. Pytorch 1.10 was used to implement all models.

**Results.** Table 2 shows the performance of CAST at the radiological grading tasks, and compares it to the state-of-the-art SpineNet model^7,19^ and a ResNet34 baseline with 3D convolutions in the first two layers (similar to the original SpineNet). To evaluate the comparative performance of 3D convolutions and attention pooling across slices, we also train the CAST encoder alone as a baseline. CAST outperforms SpineNet on the same dataset for all tasks except central canal stenosis where the difference in performance is minimal. In total average performance increases from **85.9%**$\rightarrow$**87**.**4**%. As expected, the multiple sequence model exceeds the performance of single sequence models in most tasks. The largest improvements can be seen in the endplate defect task (**82.9/87.8%** $\rightarrow$**87.2/90.7%**), disc narrowing (**76.1%**$\rightarrow$**77.4%**) and Pfirrmann grading (**71.0%** $\rightarrow$**73.0%**). The Pfirrmann grading score of the T1w-only model is far worse than the models with T2w sequences (64.5%). This matches expectations as one of the criteria of Pfirrmann grading is the intensity of the intervertebral discs in T2w sequences^38^. Overall, the improved performance is a clear indication of the benefit of considering context from multiple sequences and intervertebral discs together. We also explored grading performance for this dataset across varying magnetic field strengths, pixel spacings and imaging centres (see Supplementary Material Section 6), finding that the model is robust across a range of scan protocols.

**Table 2:** Results of the intervertebral disc grading task.

	Grading Task: # Classes:				
Model	Pfirrmann	Disc Narrowing	C.C.S.	Spondylolisthesis	
	5	4	$2^\dagger$	2	
SpineNet (T2w)^20^	71.0	76.1	95.8	95.4	
ResNet34, 3D convolutions (T2w)	70.9	76.3	93.2	95.0	
Baseline: CAST Encoder (T2w)	70.8	74.6	94.3	96.2	
CAST (T1w)	64.5	73.9	93.6	96.6	
CAST (T2w)	71.7	76.9	93.6	96.2	
CAST (T1w,T2w)	73.0	77.4	94.9	95.5	

Model	Grading Task: # Classes:				
	Endplate Defect		Marrow Change		**Average**
	Upper	Lower	Upper	Lower	
	2	2	2	2	
SpineNet (T2w)^20^	82.9	87.8	89.2	88.4	85.8
ResNet34, 3D convolutions	84.9	89.8	88.9	88.2	85.9
Baseline: CAST Encoder (T2w)	87.2	88.2	90.0	89.2	86.3
CAST (T1w)	84.7	87.0	88.4	88.7	84.7
CAST (T2w)	85.3	89.3	91.0	90.2	86.8
CAST (T1w,T2w)	87.2	90.7	90.1	89.9	87.4

The balanced accuracy for each sub-task is shown. CCS represents central canal stenosis. ^†^CCS is originally graded with 4 degrees of severity. We combine the severity classes 2-4 (mild, moderate & severe CCS) into a single class compared to class 1 (no CCS) following SpineNet.^19^. A study exploring how the performance of the grading system varies across different magnetic field strengths, pixel spacings and imaging centres is available in the supplementary material.

### VB-level classification of spinal metastases and related conditions

This section describes using CAST to detect spinal metastases and the related conditions of vertebral fractures/collapse and spinal cord compression. Instead of operating on disc levels as in the previous section, this model operates on volumes surrounding each detected vertebral body. It is also worth noting that instead of lumbar scans, the scans used in the experiments in this section are whole spine scans.

**Datasets.** We use a dataset of anonymised clinical MR scans and associated reports extracted from Oxford University Hospitals Trust. Similar to **OWS**, this dataset was extracted as part of Oxford Secondary Care Lumbar Magnetic Resonance Imaging Cohorts (OSCLMRIC), and was subject to the same Health Research Authority approval (IRAS Project ID 207858). All subjects in the dataset were over 18 and were referred for a whole spinal scan by a cancer-related specialty between April 2015 and April 2021. All patients who met these criteria were included, regardless of whether or not they had metastases, ensuring diversity in the dataset.

Instead of annotating scans with the conditions of interest, we derive labels from routine radiological reports written at the time of the scan. This offers a significant advantage in terms of speed and scalability, as it eliminates the need for a radiologist to review each image separately. Additionally, this approach requires far less clinical expertise as only a basic understanding of the relevant vocabulary is necessary to generate these labels.

However, extracting labels directly from radiological reports introduces a new challenge; dealing with ambiguities in the text. Reports can be inconclusive; e.g. “Metastases are a possibility but further investigation is required”. To deal with this, such cases are labelled as ‘unknown’ for the corresponding condition and no loss is applied (for the predictions for that condition). We also found that reports often focus on a single metastasis and other vertebrae are not fully described, particularly in cases where the radiologist is comparing findings to a previous scan. In such cases, the explicitly mentioned level is marked as positive and others are labelled as unknown. The exception is the cord compression task, where levels are always considered negative unless explicitly stated otherwise. This is because compression is a severe condition that is highly unlikely to be omitted from a report. Finally, metastatic cancer sometimes is simply reported to be widespread, with no mention of specific levels. To ensure that supervision can be extracted from these studies no vertebral level supervision is provided. Instead, the most positive prediction for the associated condition across all vertebrae in the spine is used. Therefore, our models are trained by a combination of single-instance and multiple-instance learning; single-instance in cases where exact level of the condition is indicated in the report, and multiple-instance when the exact level is not specified. Examples of vertebral bodies from each class are shown in Fig. 3. This process is described further in the supplementary material. To verify that our model’s predictions match those of custom annotations, we compare our test set results to annotations from an expert spinal surgeon (reader 1) and radiologist (reader 2).

**Training and Implementation.** The VB volumes are simply extracted by tightly fitting and slightly expanding a rectangular cuboid around each VB detection similar to the disc volume extraction Sect. "Disc-level radiological grading of spinal degenerative disorders". The resulting pixels are then resampled to $224{\hspace{-1.111pt}\times \hspace{-1.111pt}}224 {\hspace{-1.111pt}\times \hspace{-1.111pt}}S$ where *S* is the number of sagittal slices. Training details are then identical to the disc-level radiological grading task.

**Figure 5:**
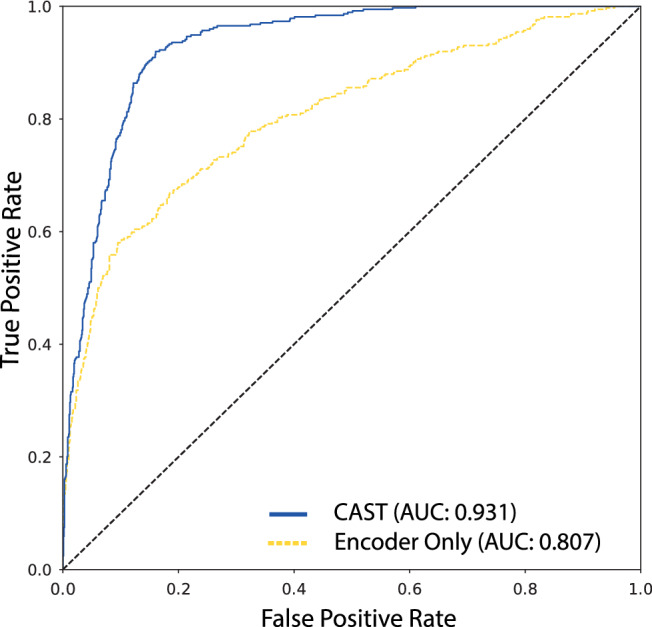
ROC curves for the metastases task for CAST and the encoder baseline. Curves for the other tasks are given in the appendix.

**Results.** Results from the cancer grading task are shown in Table 3. We find that CAST exceeds the baseline in almost all cases. In particular, there was a large improvement on the metastases grading task (AUC: **0.800**$\rightarrow$**0.931** for Reader 1’s labels), which we found to be mostly due to improved sensitivity to subtle metastases, which can be seen in Fig. 5. This is perhaps unsurprising since for subjects with multiple metastases, a clear metastasis at one level will inform borderline predictions at other levels. We note slightly depreciated performance at the compression task for reader 1 compared to the report-extracted annotations. We believe this is due to overfitting as there are relatively few compression cases in the training set.

**Table 3:** AUC scores for the three tasks with various models.

	Reader 1 Test Set Annotations		
	ROC AUC		
	Mets	Frac.	Cmprs.
Baseline	0.800	0.975	0.930
CAST	0.931	0.980	0.868

	Reader 2 Test Set Annotations		
	ROC AUC		
	Mets	Frac.	Cmprs.
Baseline	0.840	0.933	0.902
CAST	0.936	0.962	0.909

	Report-Extracted Test Set Annotations		
	ROC AUC		
	Mets	Frac.	Cmprs.
Baseline	0.934	0.901	0.955
CAST	0.944	0.902	0.918

We compare to the report-extracted annotations and also expert annotations of each image. Significant values are in [bold].

## Discussion and clinical relevance

From the above results, we can see that the presented system can provide accurate radiological grading on a per-vertebral or intervertebral disc level across a wide range of tasks, taking only a raw sagittal spinal MR scans as input. One of the key aspects of the presented system is its modularity, allowing it to be easily used by other parties as is or be extended to new clinical tasks. One could imagine, for example, adding a segmentation network along with the metastases detector, allowing the automated quantification of tumour load, which could be used to assess instability in the vertebral column. This adaptability is key considering the wide range of clinical imaging tasks a radiologist must perform with Spinal MRIs. In future work, we intend to add additional clinical tasks to the pipeline in addition to further validate the existing models on new datasets.

A potential limitation of the proposed pipeline is the input. Currently, only sagittal MRI scans are used, however it would be beneficial to incorporate other views of the spine such as coronal and axial, which are commonly used in clinical practice. This could provide complementary information to improve performance. Additionally, incorporating other clinical data such as patient history and previous scans may contextualize the imaging findings for more holistic assessments.

The presented system is a research tool and has not yet been sufficiently validated for use in clinic. However, we believe it is an important step towards realising fully automated radiological grading of spinal MRIs in clinical settings. We envisage several uses for such a system: Firstly, it could provide a rapidly available second opinion for clinicians evaluating spinal MRIs, lessening the risk of missed signs and reducing radiologist workload. Alternatively, it could be used for triaging, informing radiologists of the most urgent scans to read from a longer list. Finally, it could be of great benefit in large-scale drug trials studies involving spinal imaging, or epidemiological studies of spinal disorders, where thousands of scans must be reviewed and manual annotation by experts would be too slow and expensive.

## Summary

In this paper, we present an end-to-end approach to analysing spinal MR scans. We began by using vector field regression to detect and label vertebrae across a range of common MR sequences and fields-of-view and find it improves on others methods across several clinical datasets. The second part of the paper introduces the Context-Aware Spinal Transformer, an architecture for performing spinal disorder classification on vertebral bodies/intervertebral discs. Similar to radiologists, CAST explicitly incorporates context from neighbouring vertebrae and other MR sequences when making classification decisions. We train CAST on two spinal disease classification problems: performing radiological grading on intervertebral discs and detecting spinal metastases, cord compression and fractures in vertebral bodies. Finally, the resulting pipeline’s code and trained models are made available open-source to computer vision and back pain researchers.

## Data collection authorization

The Genodisc dataset was curated by the Genodisc consortium with approval from the UK Research Ethics Committee 09/H0501/95 as part of The European Union Health Project on Genes and Disc Degeneration called ‘Genodisc’ (FP7 Health 2007A Grant Agreement No. 201626). Equivalent IRB/REC approvals were obtained in each recruiting country.

The Oxford Whole Spine (OWS) dataset and spinal metastases datasets are part of Oxford Secondary Care Lumbar Magnetic Resonance Imaging Cohorts (OSCLMRIC) which is a Health Research Authority approved study (IRAS Project ID 207858). The University of Oxford is the sponsor of this research, in keeping with the requirements of the UK Policy Framework for Health and Social Care Research 2017. Health Research Authority approval for receipt and analysis of anonymised retrospective patient data was received in 2016 (project reference 207858) to assist in the development of an image analysis methodology to analyse clinical MRI studies in subjects with low back pain syndromes and asymptomatic controls. PID 12139 Protocol Number 12139. Date/version 23/08/2016; v9.0; Minor amendments (to increase scope of recruitment and duration) were requested 18th March 2019) All the subjects in this report had been recruited before this date. IRAS Project ID: 207858 REC Reference: 16/HRA/4532 Short Study.

### Supplementary Information

Supplementary Information.

## Data Availability

The code and trained models weights described in this paper are made publicly available at the following URL: https://github.com/rwindsor1/SpineNet. The Genodisc dataset can be accessed by request to the Genodisc consortium. OWS and the spinal metastases dataset were extracted and anonymised from local hospital systems under a contract which explicitly forbids data sharing with third parties for patient privacy reasons and thus cannot be made available. The Genodisc dataset was curated by the Genodisc consortium with approval (including waiving informed consent) from the UK Research Ethics Committee (REC Reference: 09/H0501/95) as part of The European Union Health Project on Genes and Disc Degeneration called ‘Genodisc’ (FP7 Health 2007A Grant Agreement No. 201626). Equivalent IRB/REC approvals were obtained in each recruiting country. The Oxford Whole Spine (OWS) dataset and spinal metastases datasets are part of Oxford Secondary Care Lumbar Magnetic Resonance Imaging Cohorts (OSCLMRIC) which is a Health Research Authority (REC Reference 16/HRA/4532) approved study (IRAS Project ID 207858).The University of Oxford is the sponsor of this research, in keeping with the requirements of the UK Policy Framework for Health and Social Care Research 2017. This includes approval for receipt and analysis of anonymised retrospective patient data with waived informed consent, which was received in 2016 to assist in the development of an image analysis methodology to analyse clinical MRI studies in subjects with low back pain syndromes and asymptomatic controls.
