# Supplementary material for: Automated detection, labelling and radiological grading of clinical spinal MRIs
Source: Sci Rep. 2024 Jul 1;14:14993. doi: 10.1038/s41598-024-64580-w (PMC11217300; doi:10.1038/s41598-024-64580-w)
Supplement: Supplementary file 1 — Supplementary Information. [file 41598_2024_64580_MOESM1_ESM.pdf]

Automated Detection, Labelling and Radiological Grading of Clinical Spinal MRIs - Supplementary Material

Rhydian Windsor[1,*], Amir Jamaludin[1], Timor Kadir[1,2], and Andrew Zisserman[1]

[1]Visual Geometry Group, Department of Engineering Science, University of Oxford
[2]Plexalis
[*]rhydian@robots.ox.ac.uk

ABSTRACT

This is the supplementary material provided for “Automated Detection, Labelling and Radiological Grading of Clinical Spinal MRIs" - under review as part of the *Scientific Reports* collection “Machine learning applications in medical image analysis".

1 Vertebra Detection + Labelling Datasets Breakdown

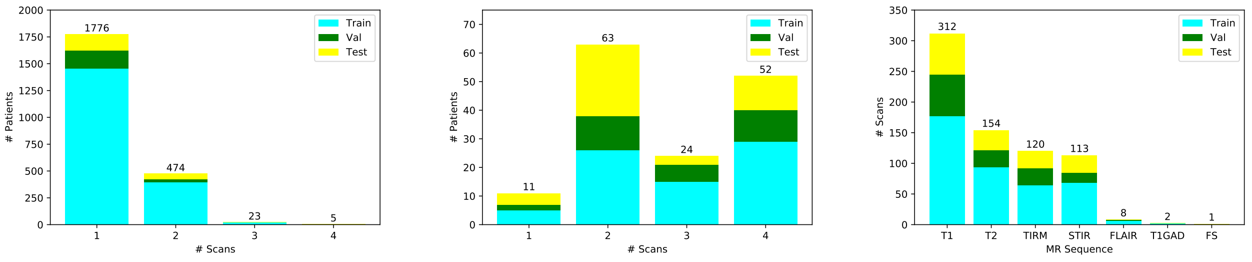

(a) Scans per patient in Genodisc. **(b)** Scans per patient in OWS. **(c)** MR sequence types in OWS.

Supplementary Figure S(1). Breakdown of scan types in both OWS (196 patients, 719 scans) and Genodisc (2279 patients, 2819 scans). Both datasets are split 80:20:20% down the patient line.

2 Splitting Larger Scans Into Patches

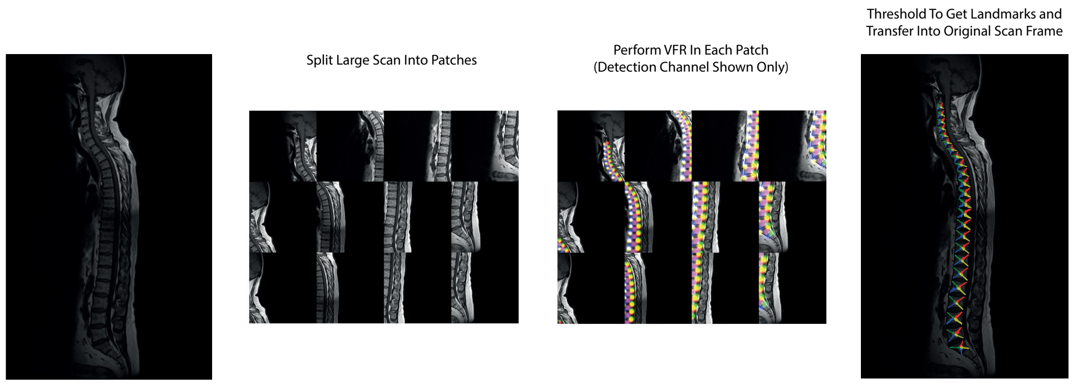

Supplementary Figure S(2). The process of splitting larger scans into patches for VFR. Detections of landmarks and grouping vectors are made patchwise, then transformed back into the frame of the scan, in which grouping is performed.

3 Spinal Metastases Dataset

Split	Patients	Studies	Total	Vertebral Bodies								
				Metastases			Fractures			Compression		
				+	−	?	+	−	?	+	−	?
Training	258	558	12459	829	6099	5531	216	12124	119	90	12369	0
Validation	33	63	1391	81	829	482	25	1360	7	15	1377	0
Test (Reports)	32	66	1430	92	646	692	22	1408	0	11	1419	0
Test (Reader 1)	32	66	1430	374	1056	0	46	1384	0	31	1399	0
Test (Reader 2)	32	66	1430	442	988	0	57	1373	0	15	1415	0

Supplementary Table S(1). The dataset used to train SCT to detect metastases, fractures and compression. The first-three rows indicate the number of labels extracted from free-text reports for the training, validation and test splits. The bottom row indicates the same test dataset with each vertebra independently annotated by an expert. Vertebra are labelled as positive (+), negative (−), or unknown (?). Note that the expert annotators were asked to make their best guess in cases of uncertainty.

4 Robustness To Pathology

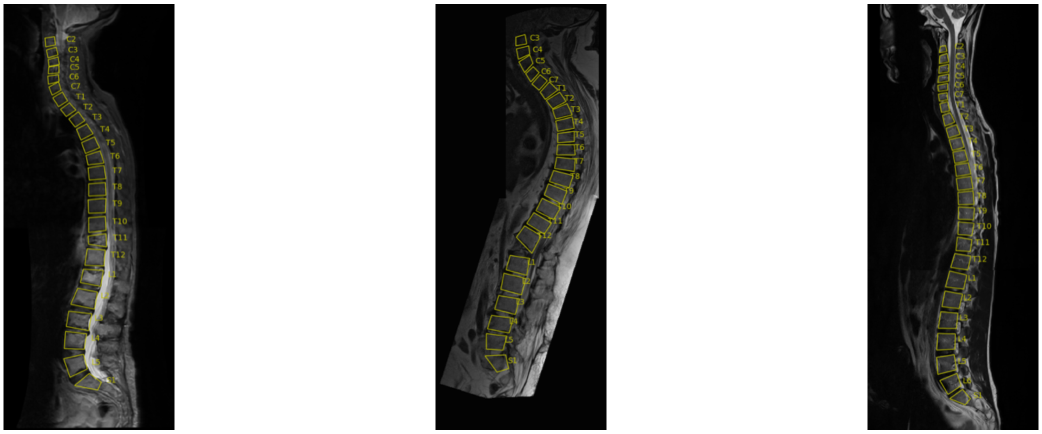

(a) Collapsed T11 Vertebra **(b)** Hemivertebra **(c)** Extra (Transitional) Vertebra

Supplementary Figure S(3). Robustness to realtively common anatomical variations - collapsed vertebrae,transitional vertebrae and hemivertebrae. Note that while the model does not detect the hemivertebra, it is able to adapt to the sudden change in spinal curvature. The detection model can also be used to measure spinal in cases of scoliosis, or hyperlordosis/kyphosis.

5 Radiological Gradings in Genodisc

The **Genodisc** dataset consists of 2635 subjects diagnosed with back pain. Of the 2635, only 2079 were assessed and scored by a radiologist and of those 2079, only 2009 possess readable scans. Each scan contains up to six lumbar discs per subject, with the majority having a complete field-of-view of the lumbar region and including six discs. In total there are 12018 discs, from 2009 subjects, each possessing several radiological gradings. The dataset was collected by the Genodisc Project in 2009 (`www.physiol.ox.ac.uk/genodisc`). **Genodisc** recruited "patients who seek secondary care for their back pain or spinal problem", and recorded their MR scans and clinical information. The scans were sourced from multiple centres: England (Oxford, Oswestry, Kettering), Hungary (Budapest), Slovenia (Ljubljana) and Italy (Milan). They came from different machines, and were acquired with a variety of acquisition protocols following the clinical standard of each centre. Because of this, differences in the scans can be quite noticeable e.g. the slice thickness of the sagittal scans may vary substantially. All the subjects were scanned in the supine position, lying on their backs. 54.6% of the subjects are females. Some details of the subjects and their scans can be see in Table 2.

A set of scans, or each series, from a subject contains multiple type of scans: T1-weighted axial, T1-weighted sagittal, T2-weighted axial and T2-weighted sagittal. The axials scan are normally only available for the bottom three lumbar discs,

	Mean	Median	Range
**Age**	50.1	50.0	14.0 – 87.0
**Weight**	77.8	80.0	43.0 – 130.0
**Field Strength (Tesla)**	1.3	1.5	0.2 – 3.0
**Sagittal Slice Thickness (mm)**	4.1	4.0	1.2 – 6.0
**Sagittal Pixel Spacing (mm)**	0.7	0.6	0.3 – 1.6
**Sagittal Slice Gap (mm)**	4.8	4.7	0.0 – 8.8
**Sagittal Slice Count**	12.3	11.0	8.0 – 25.0
**Axial Slice Thickness (mm)**	4.5	4.0	1.3 – 10.0
**Axial Pixel Spacing (mm)**	0.5	0.5	0.2 – 1.5
**Axial Slice Gap (mm)**	5.3	5.0	0.0 – 46.8
**Axial Slice Count**	12.5	12.0	9.0 – 22.0

Supplementary Table S(2). Subject & Scan Details – Genodisc. Details of the subjects and scans in the **Genodisc** dataset that possess MRIs and were read by a radiologist.

L3-L4, L4-L5, and L5-S1. The slices of the axial scans can be obtained in two different orientations (see Figure 4): (i) in blocks where all axial slices are parallel to each other, or (ii) per disc basis where slices pertaining to each disc are aligned to be parallel to the disc orientation. T2-weighted sagittal scans are used the most in this thesis as most radiological gradings for the spine can be assessed via T2-weighted sagittal scans, and these often emphasize clinically popular gradings compared to T1-weighted scans.

The scans were assessed by a single expert spinal radiologist, Professor Iain McCall, who produced a range of global, i.e. the whole spine, and local, i.e. per disc, gradings. Overall, there are 16 different radiological scores where each one has its own specific scale of measure as shown:

1. **Pfirrmann grading [S]:** 1 – 5 (?)
2. **Disc narrowing [S]:** 0 – 3 (normal, slight, moderate, severe)
3. **Annular tears (HIZ) [S & A]:** 0 – 2 (absent, present, contiguous)
4. **Anterior disc bulging [S & A]:** 0 – 3 (normal, slight, moderate, severe)
5. **Posterior disc bulging [S & A]:** 0 – 3 (normal, slight, moderate, severe)
6. **Disc herniation [S & A]:** 0 – 3 (normal, slight, moderate, large)
7. **Location of herniation [S & A]:** Central (**C**), Posterolateral R or L (**PLR** or **PLL**), Foraminal R or L (**FR** or **FL**), and Other (**O**)
8. **Type of herniation [S & A]:** 1 – 3 (protrusion, extrusion, sequestration)
9. **Nerve root compression (herniation) [A]:** 0 – 3 (none, touching, displaced, compressed)
10. **Foraminal stenosis [S & A]:** 0 – 3 (absent, mild, moderate, severe)
11. **Nerve root compression (foraminal) [A]:** 0 – 3 (none, touching, displaced, compressed)
12. **Central canal stenosis [S & A]:** 0 – 3 (absent, mild, moderate, severe)
13. **Spondylolisthesis [S]:** 0 – 4 (0%, 25%, 50%, 75%, 100% of vertebral body sagittal plane width)
14. **Endplate defects [S]:** 0 – 3 (normal, slight, moderate, severe)
15. **Modic changes [S]:** 1 – 3 (?)
16. **Facet joint arthropathy [S & A]:** 0 – 3

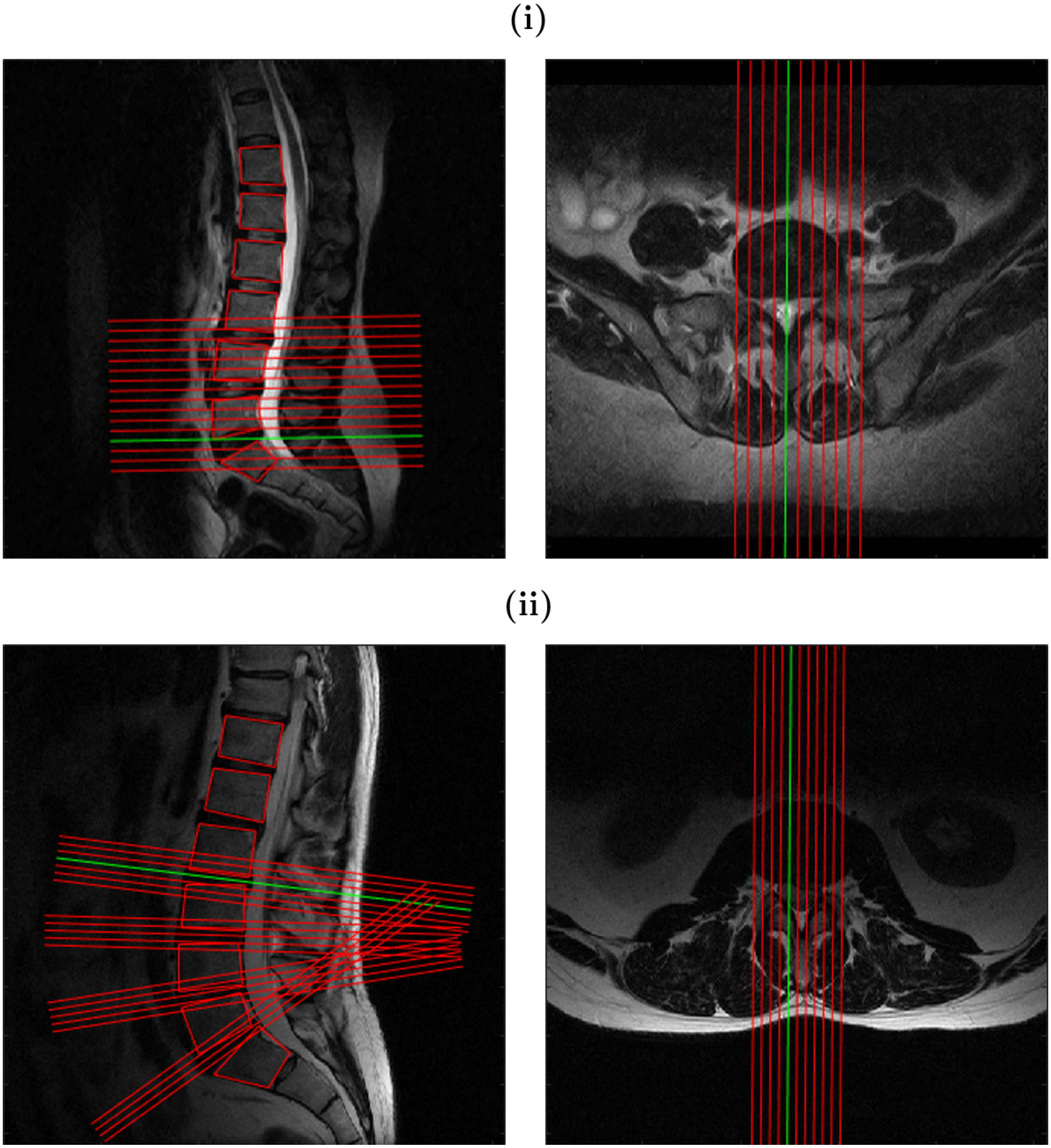

Supplementary Figure S(4). Examples of T2-weighted Axial & Sagittal Scans. The two series of scans have different orientations of axial slices here overlaid on top of each corresponding mid-sagittal slice in the same series. **(i)** Axial slices oriented in blocks which is more common in **Genodisc**, and **(ii)** axial slices oriented in parallel to each disc, this case possesses axials of four lumbar discs instead of three which is the common amount for other subjects in **Genodisc**.

Most of the scores were acquired by assessing both sagittal and axial scans **[S & A]**, five needed only sagittal **[S]**, and two needed only axial **[A]** scans. Out of these 16 gradings, we experimented on the following gradings: **Pfirrmann grading** (Figure 7), **disc narrowing** (Figure 8), **central canal stenosis** (Figure 9), **spondylolisthesis** (Figure 10), **disc hernation** (Figure 15), **endplate defects** (Figure 11), **modic changes** or **marrow changes** (Figure 12). **Endplate defects** and **marrow changes** are further divided into a classification of lower and upper endplate gradings such that the two gradings can be viewed as four separate binary classification tasks. Figure 5 gives the grade distribution of the radiological gradings. Since severe gradings are extremely rare, all the gradings used in our experiments are binarised to just two classes i.e. normal and abnormal except for **Pfirrmann grading** and **disc narrowing**.

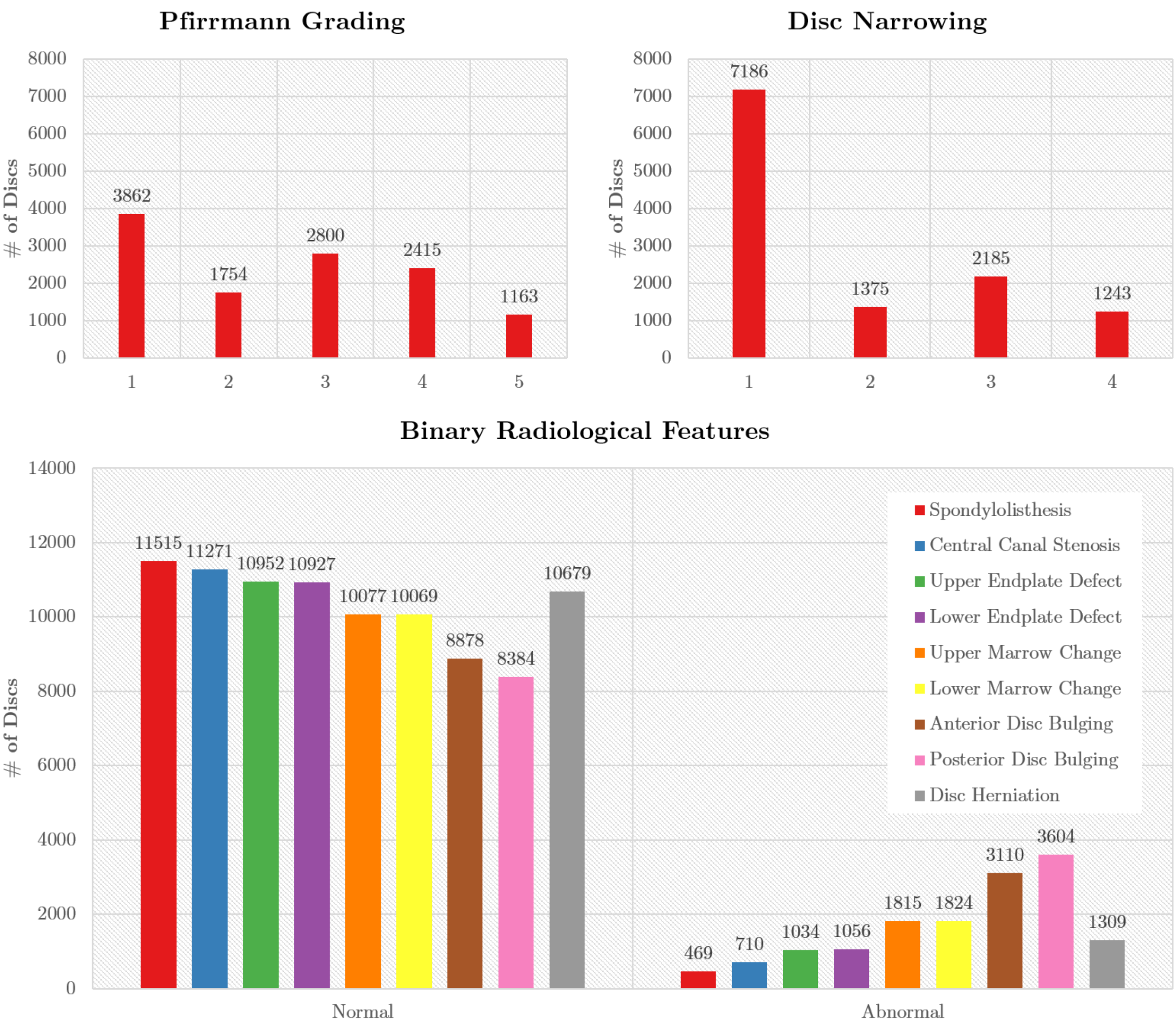

Supplementary Figure S(5). Distribution of The Radiological Gradings (per disc). Both **endplate defects** and **marrow changes** have two separate gradings, one each for both the upper and lower endplate regions. Note, there is a total of 12018 discs but since there are missing labels (independent of grading), the totals of labelled discs shown in the table for each grading are different. **Pfirrmann grading** and **disc narrowing** are multi-class while the other gradings are binary.

6 Robustness To Scan Parameters

To be useful in as many scenarios as possible, it is important that the proposed automated grading system is robust to changes in scanning protocols such as magnetic field strength and pixel spacing. To explore this, Figure 6 shows the performance of

the T2 sagittal grading model on stratified subsets of the **Genodisc** test set. Balanced accuracy measured across four tasks: *Pfirrmann Grading, Disc Narrowing, Marrow Changes* and *Foraminal Stenosis*. Since some strata had limited postive cases, the multiclass tasks are reduced to binary classification tasks with approximately similar numbers of positives and negatives (*Pfirrmann* is split as classes [1,2] versus [3,4,5] and *Disc Narrowing* as [1] versus [2,3,4]). The empirical distributions of both field strength and pixel spacing in our test dataset is also shown. We find that the concordance of the model's predictions with radiologist gradings increases slightly correlated with magnetic field strength and anti-correlated with pixel grading, however this appears to vary from task to task. The models performance across these tasks also does not appear to vary significantly across the different imaging centres in the test dataset.

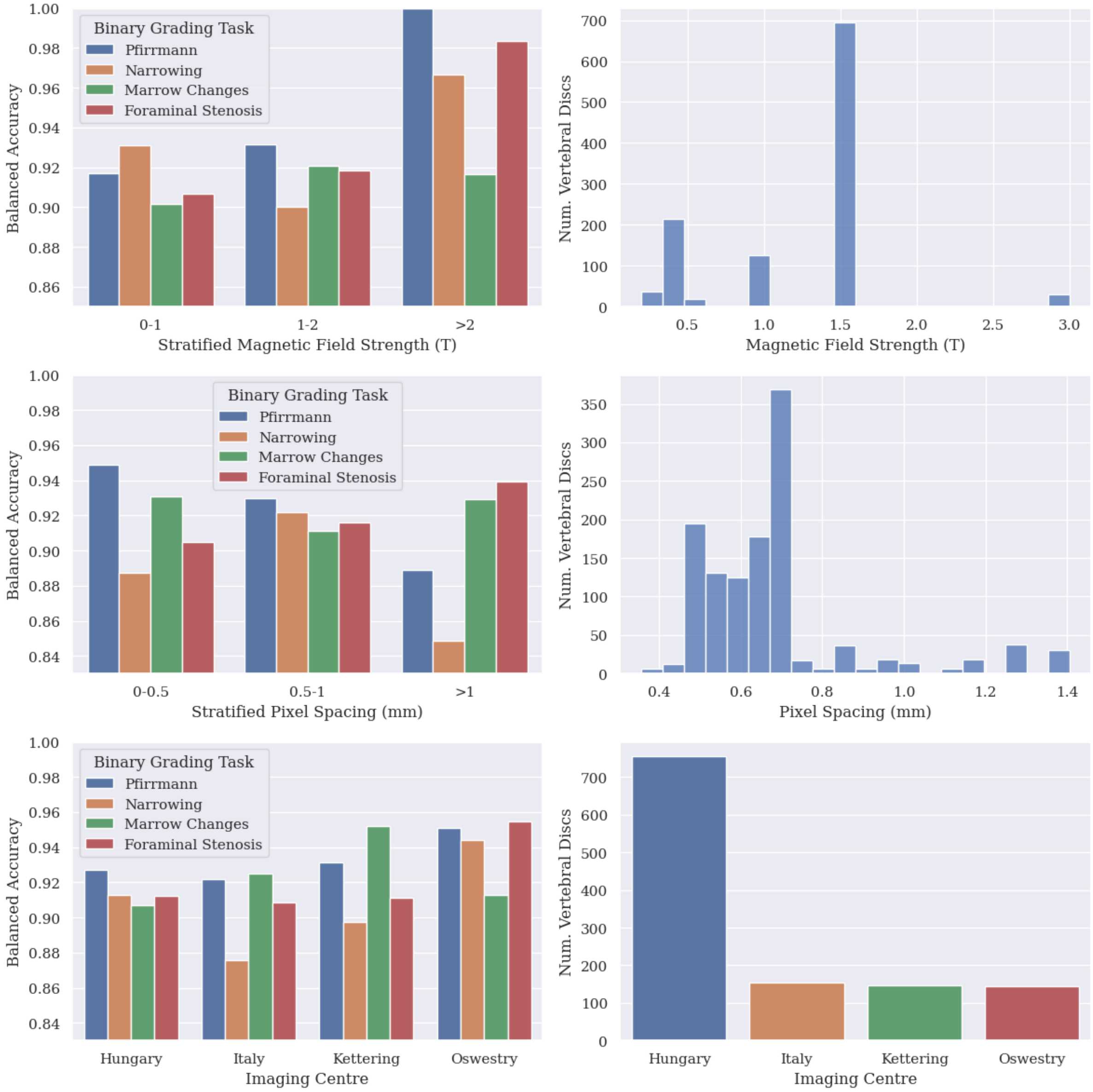

Supplementary Figure S(6). Grading performance stratified by magnetic field strength, sagittal pixel spacing and the imaging centre at which the scan was conducted, shown for the Genodisc test set. The distribution of these scan parameters in the test set is shown in the right-hand plots.

7 Example Radiological Gradings

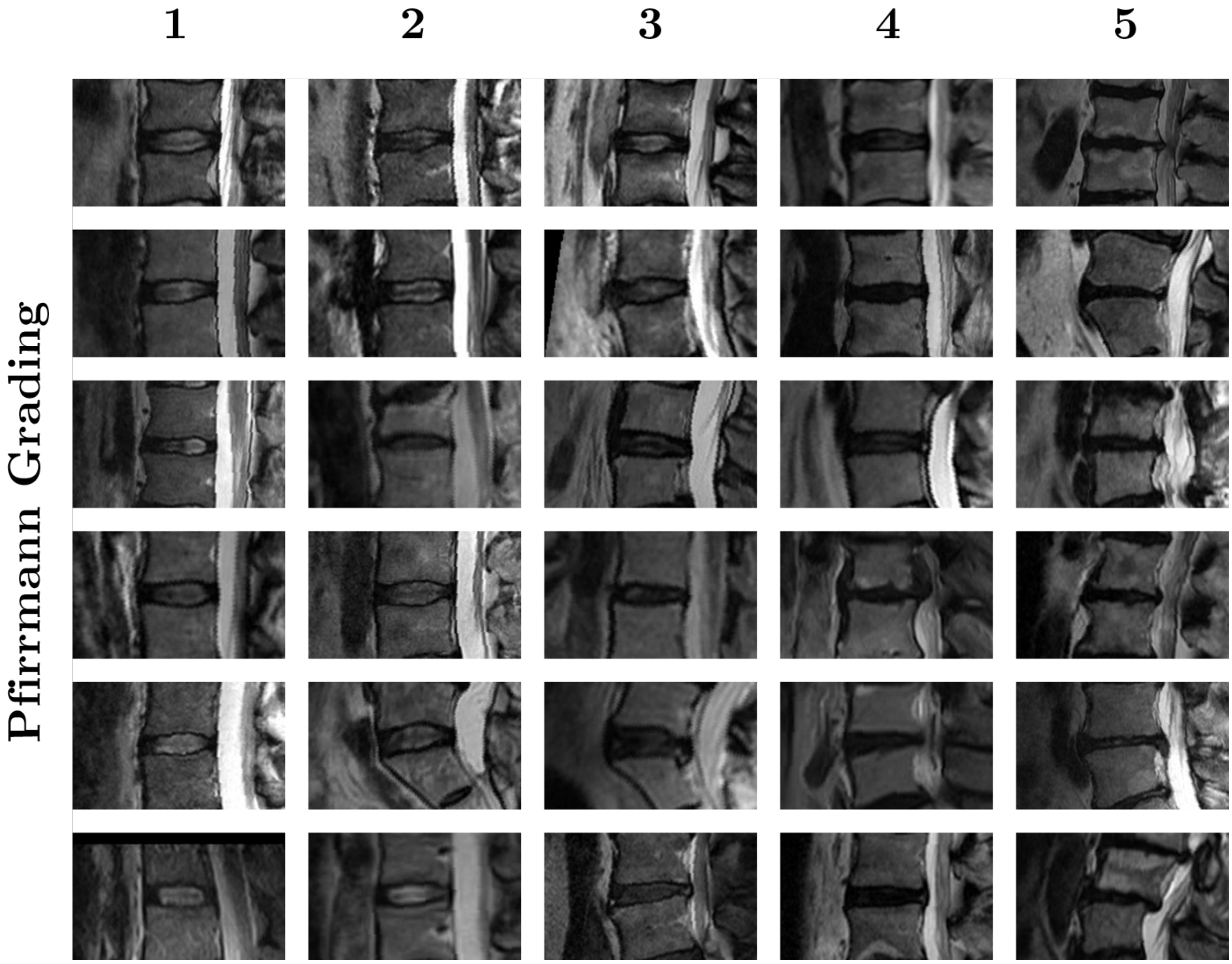

Supplementary Figure S(7). Examples of Pfirrmann Grading. **Pfirrmann grading** is a grading system of disc degeneration using criteria of disc signal heterogeneity, brightness of the nucleus and disc height; 5 grades.

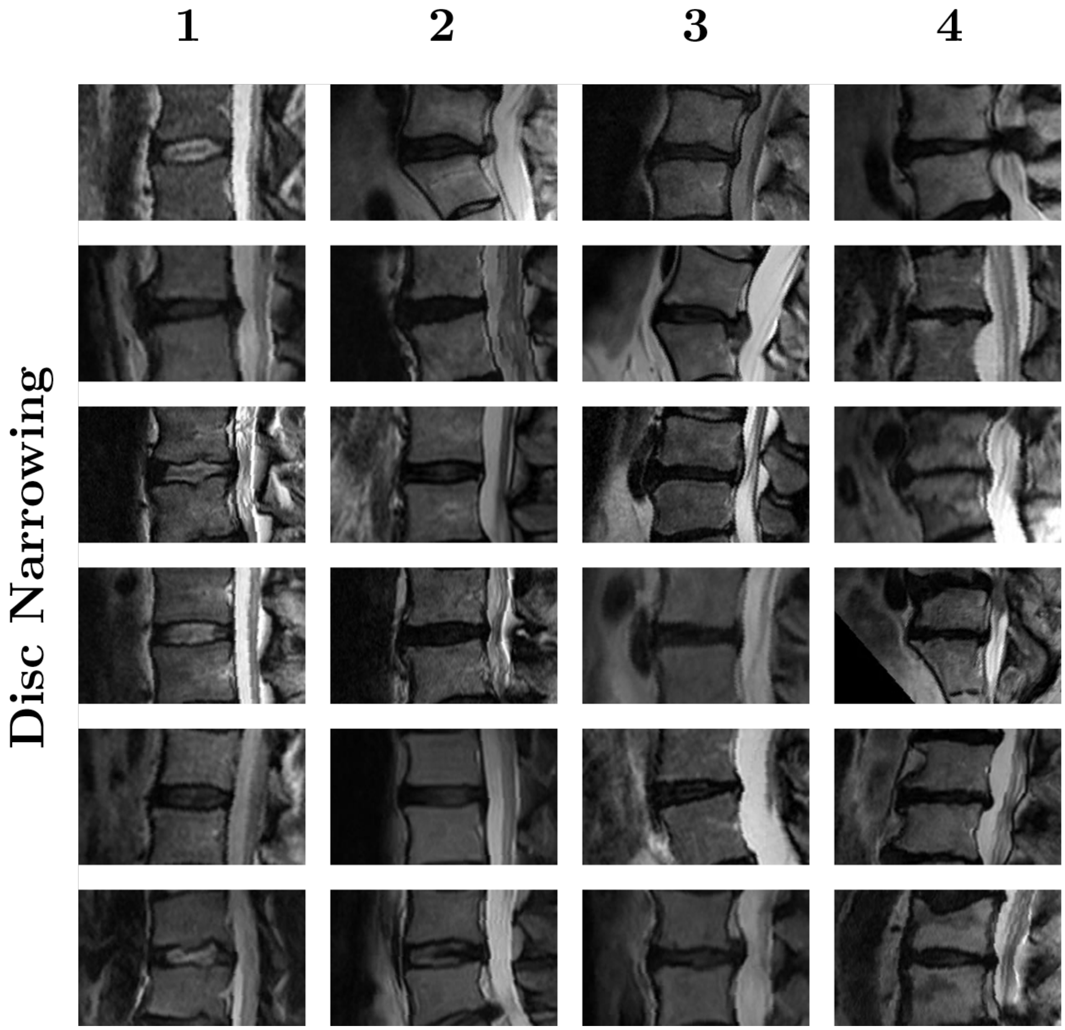

Supplementary Figure S(8). Examples of Disc Narrowing. **Disc narrowing** is defined as a multi-class measurement of the disc heights; 4 grades.

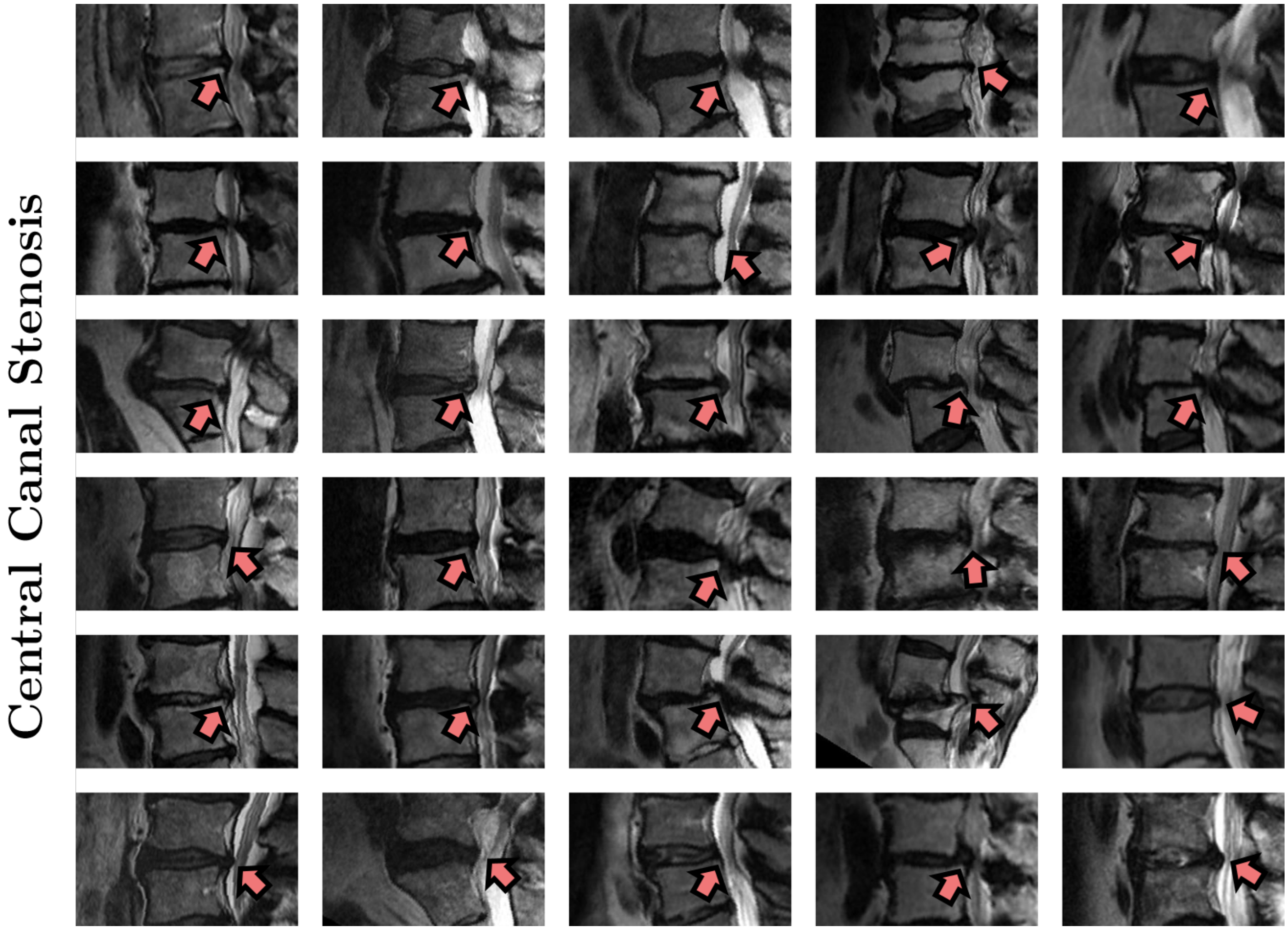

Supplementary Figure S(9). Examples of Central Canal Stenosis. **Central canal stenosis** is the constriction of the central canal in the region adjacent to each intervertebral disc. The grading is based on assessment of both sagittal and axial images. We only look at a binary 'presence' or 'absence' of stenosis in the sagittal scans.

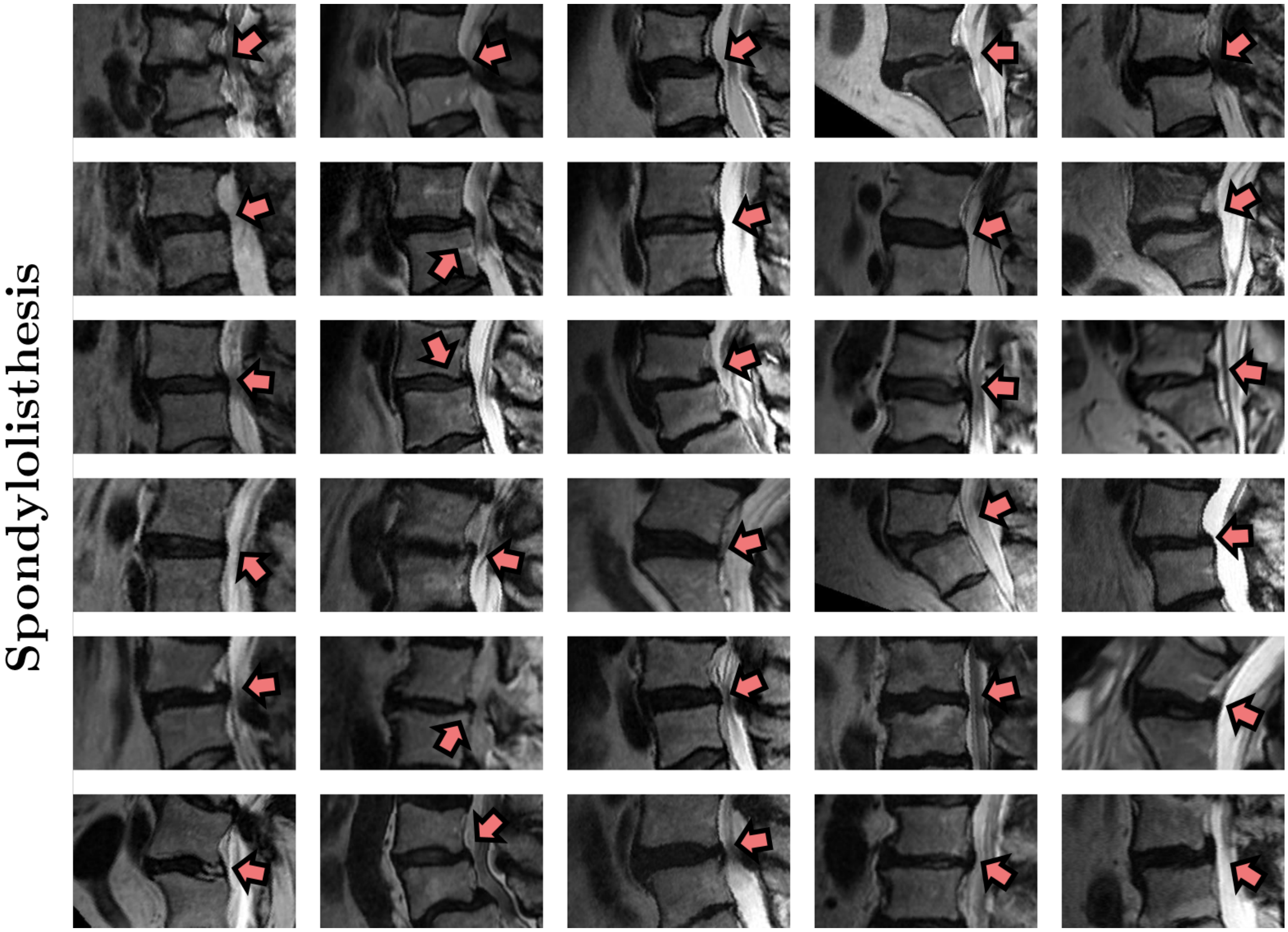

Supplementary Figure S(10). Examples of Spondylolisthesis. **Spondylolisthesis** is a binary measure of the vertebral slip; i.e. is the pair of vertebrae above and below a disc in-line or has it slipped?

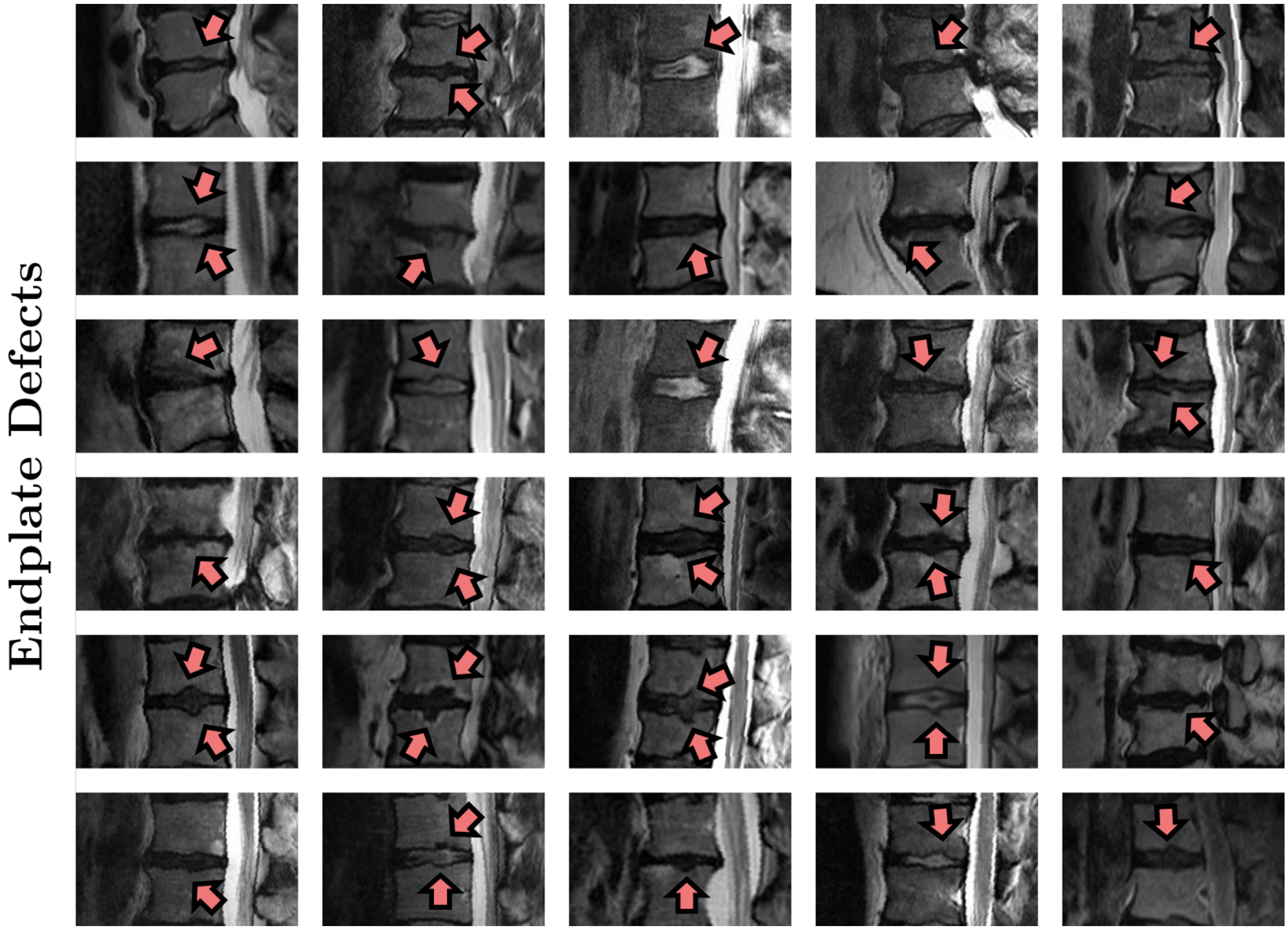

Supplementary Figure S(11). **Examples of Endplate Defects**. **Endplate defects** are deformities of the endplate regions, both upper and lower, with respect to the intervertebral disc.

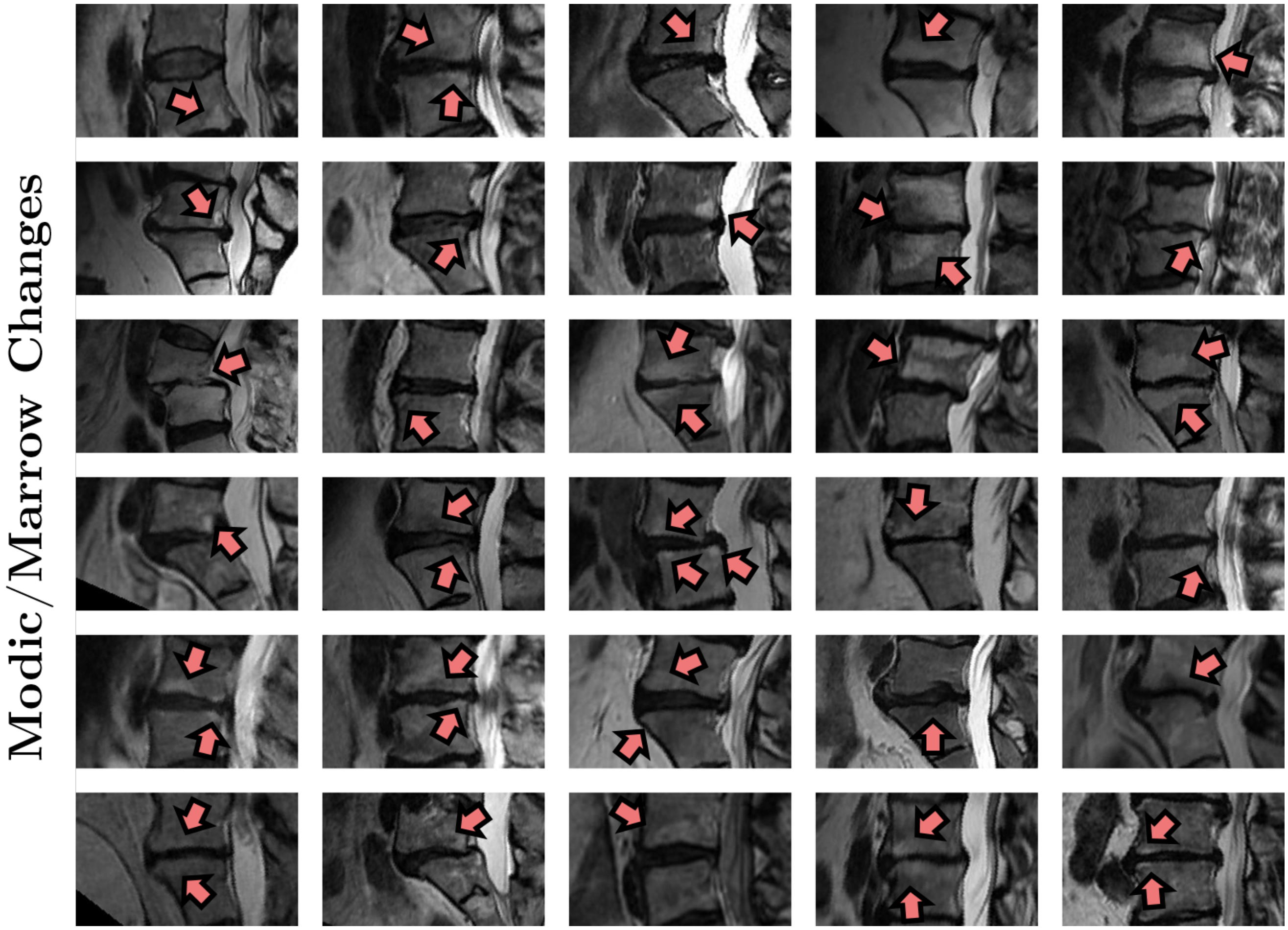

Supplementary Figure S(12). Examples of Marrow Changes. **Marrow changes** are visible signal variations that occur in the vertebral endplate regions, upper or lower. It can show up as both hyper- or hypo-intense signals of the vertebral bodies.

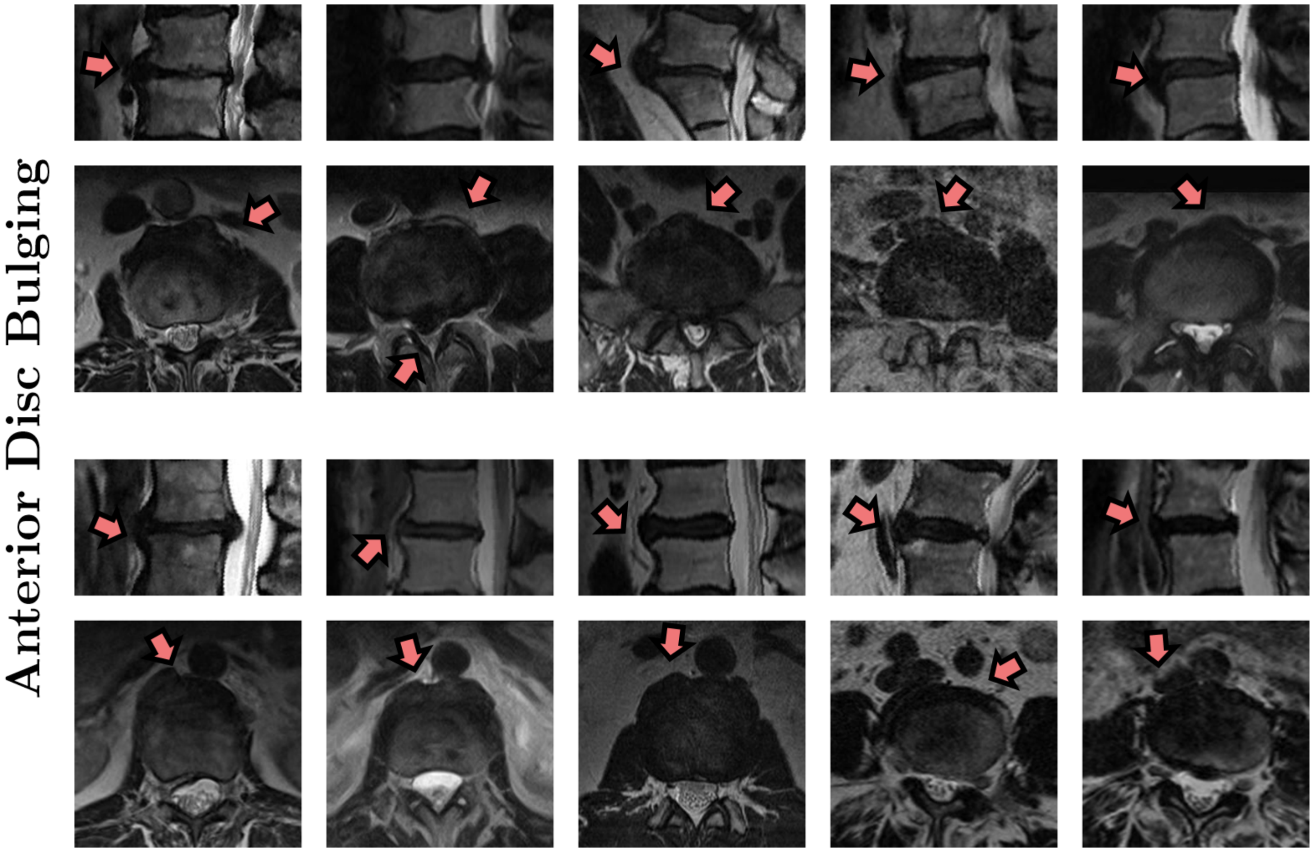

Supplementary Figure S(13). Examples of Anterior Disc Bulging. **Anterior disc bulging** is the protrusion of the frontal part of the intervertebral disc. Normally graded by viewing both sagittal and axial scans. The top row is the sagittal scan and the bottom is the axial view of the same disc.

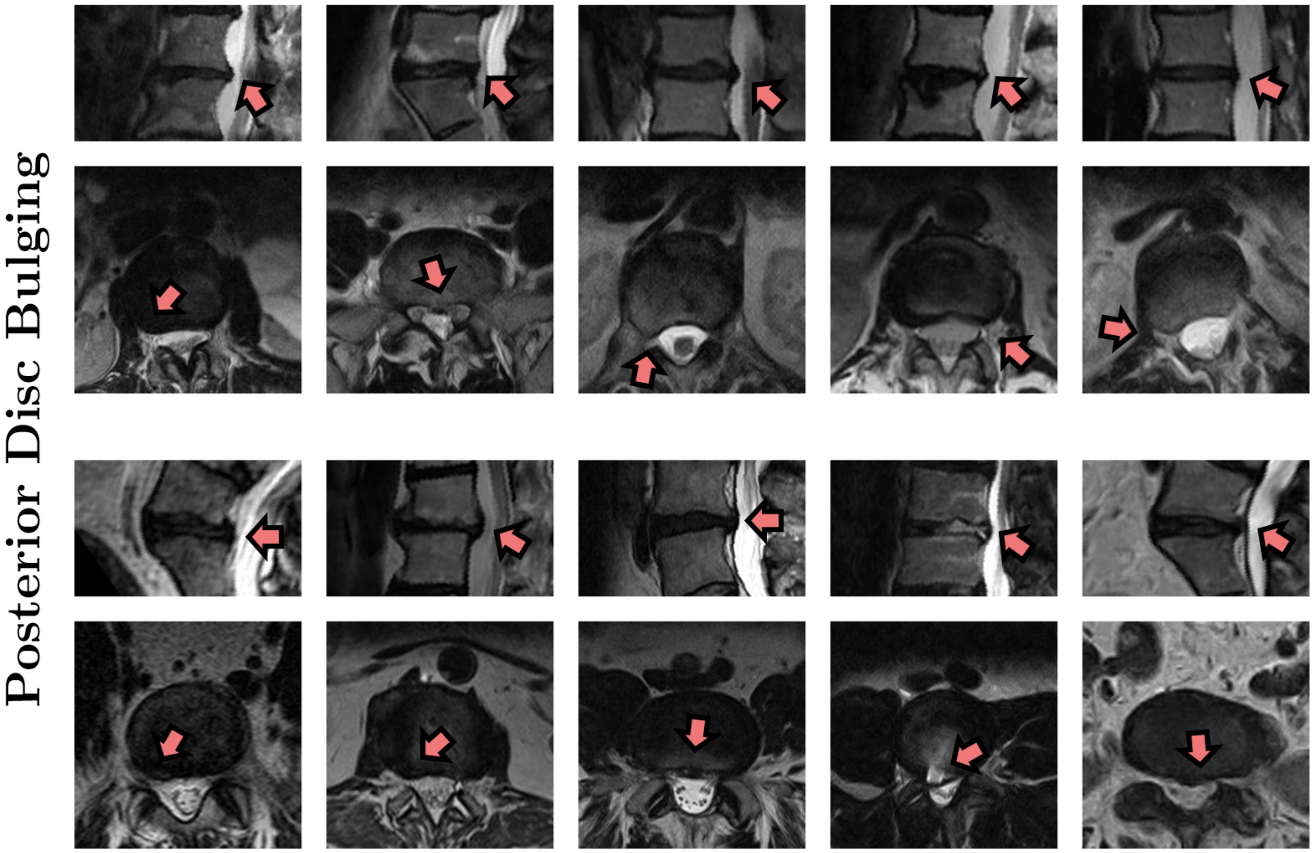

Supplementary Figure S(14). **Examples of Posterior Disc Bulging**. **Posterior disc bulging** is the protrusion of the back of the intervertebral disc. Normally graded by viewing both sagittal and axial scans. The top row is the sagittal scan and the bottom is the axial view of the same disc.

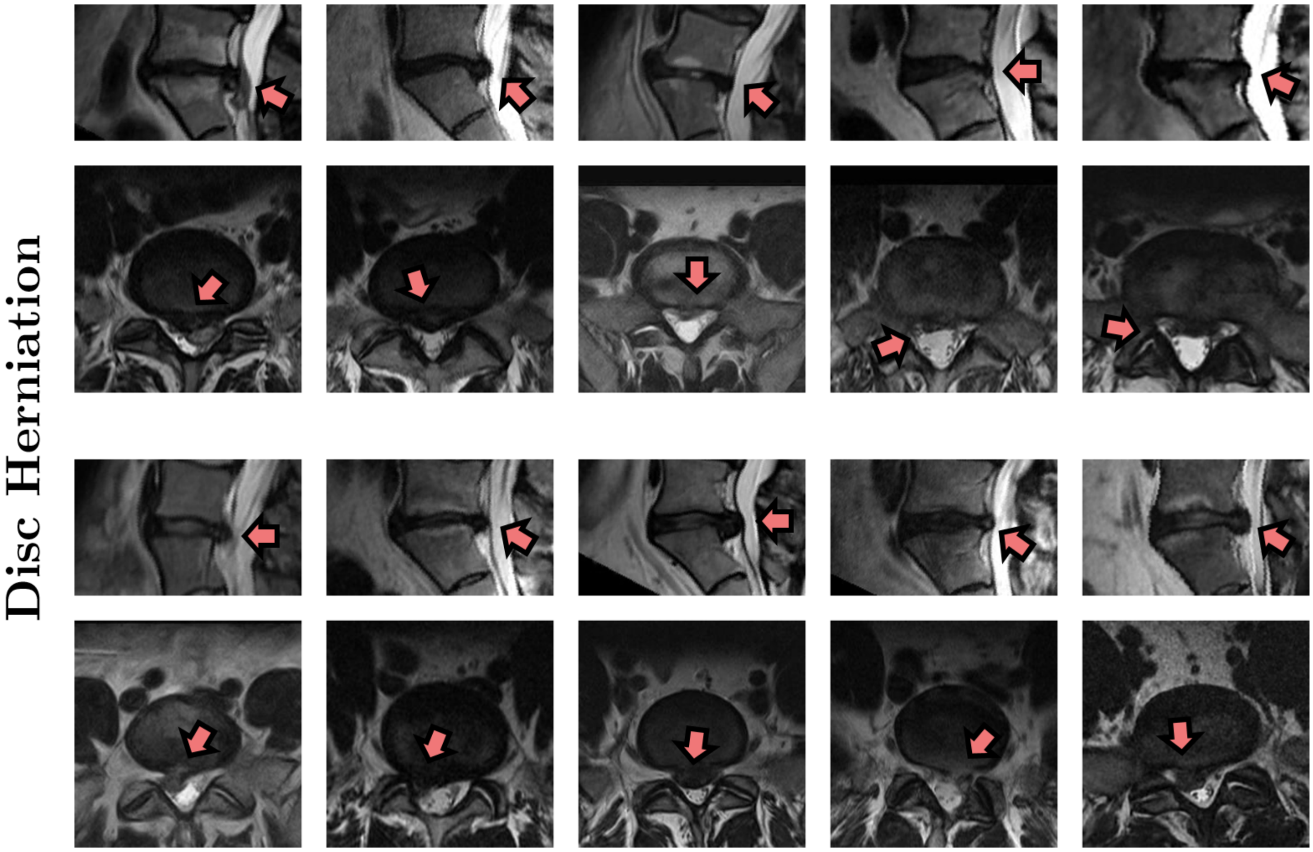

Supplementary Figure S(15). **Examples of Disc Herniation**. **Disc herniation** is similar to **posterior disc bulging** in Figure 14 with a minor distinction in that the annulus of the disc is ruptured and there is displacement of disc material in a herniated case. Normally graded by viewing both sagittal and axial scans. The top row is the sagittal scan and the bottom is the axial view of the same disc.
